# Composition templating for heterogeneous nucleation of intermetallic compounds

**DOI:** 10.1038/s41598-024-59709-w

**Published:** 2024-04-18

**Authors:** Zhongping Que, Yun Wang, Zhongyun Fan, T. Hashimoto, X. R. Zhou

**Affiliations:** 1 Brunel Centre for Advanced Solidification Technology (BCAST), Brunel University London, Uxbridge, Middlesex UB8 3PH UK; 2 School of Materials, University of Manchester, Manchester, M13 9PL UK

**Keywords:** Interfacial segregation, Fe, AlB_2_, Heterogeneous nucleation, Grain refinement, Engineering, Materials science, Physics

## Abstract

Refinement of intermetallic compounds (IMCs) through enhancing heterogeneous nucleation during casting process is an important approach to improve the properties of aluminium alloys, which greatly increases the economy value of recycled Al-alloys. However, heterogeneous nucleation of IMCs is inherently more difficult than that of a pure metal or a solid solution. It requires not only creation of a crystal structure but also the positioning of 2 or more different types of atoms in the lattice with specific composition close to that of the nucleated IMCs. Previous understanding on heterogeneous nucleation is based on structural templating, usually considering the small lattice misfit at the interface between the nucleating solid and substrate. In this work, we proposed a hypothesis and demonstrated that composition templating plays a critical role in heterogeneous nucleation of IMCs. The experimental results revealed that segregation of Fe atoms on the AlB_2_ surface, i.e., the Fe modified AlB_2_ particle, provides the required composition templating and hence enhances heterogeneous nucleation of α-Al_15_(Fe, Mn)_3_Si_2_, resulting in a significant refinement of the α-Al_15_(Fe, Mn)_3_Si_2_ particles in an Al-5 Mg-2Si-1.0Mn-1.2Fe alloy.

## Introduction

Recycling plays an important role in circular economy, helps building up the green manufacture and reduces the environmental damage^1–5^. Aluminium (Al) recycling saves up to 95% of the energy and reduces 97% of greenhouse gas emission required from primary aluminium production^1,2^. The traditional process for aluminium recycling requires large investment on the scrap aluminium sorting, and the extra cost in adding alloying elements and removing the unwanted ones to meet the customers specifications. Although the recycled aluminium scraps can be treated with refined sorting, purifying and/or dilution, these Al-alloys still lost some mechanical properties after recycling and became lower valued Al-alloys^6–10^. The key factor of the deteriorated mechanical properties of the recycled Al-alloys is due to the impurities such as Fe and Si, etc. involved in the recycling aluminium resource and accumulated with the increasing recycling cycles^11–13^. Fe is one of the critical and unavoidable impurities in Al-alloys that plays an important role in determining the mechanical properties especially the ductility. Fe has a very low solid solubility in aluminium, which leads the formation of Fe intermetallic compounds (IMCs) in large size^14^. The Fe-IMCs with variable compositions and constitute alloy elements have been reported with different crystal structures and morphologies^14–20^. These Fe-IMCs, especially those with needle-like or plate-like morphology, have detrimental effects on the mechanical properties of Al-alloys. However, due to the sensitivity of the solidification conditions, the formation of the Fe-rich IMCs is complicated and competitive, and therefore it is hard to predict by phase diagram simulation. Refinement of the Fe-rich IMCs has been a problem that desperately needs to be solved to enhance the mechanical properties of recycled Al-alloys.

De-ironing is the common way when the Fe concentration is high to form the primary Fe-IMCs^12,21^. Usually, the concentration of Fe can be reduced down to 0.7 wt % in recycled Al-alloys^22^. The Fe-IMCs formed in the Al-alloys with Fe concentration lower than 0.7 wt.% are mainly existing in the eutectic structure. The effective way so far in minimizing the detrimental effect of the remaining Fe in recycled Al-alloys is modifying the morphology, from angular to compact for instance. The Fe-IMCs such as α-Al_15_(Fe, Mn)_3_Si_2_ which has a Chinese script morphology is less harmful compare to the β-Al_4.5_FeSi that has a plate-like morphology. Usually, formation of α-Al_15_(Fe, Mn)_3_Si_2_ can be promoted by adding Mn with the atomic ratio lower than 2:1 of Fe:Mn in Al-alloys. However, the modified Fe-IMCs are still large-sized and undesirable.

Previous studies were mainly concentrating on the approaches such as thermal control^23^ and chemical methods^24–26^ to achieve grain refinement for pure metals and alloys such as aluminium. So far, one of the most successful ways to grain refining Al-alloys during the solidification process is adding grain refiner prior to casting. The mechanism of this approach was understood in terms of the supplied potent particles for heterogeneous nucleation and the alloying elements for growth restriction^27–29^. However, there is a big difference in heterogeneous nucleation between a pure metal or alloy and intermetallic compound, such as between α-Al and Fe-rich IMCs. The difference is the number of the types of constitute alloying elements. Heterogeneous nucleation of a pure metal such as Al involves only one element (probably also some minor impurities). Therefore, once the nucleation substrate that has a small lattice misfit with the nucleating solid is introduced, heterogeneous nucleation can be enhanced. However, heterogeneous nucleation of Fe-IMCs requires not only the creation of a crystal structure similar to the IMC, but also the positioning of multiple types of atoms in the lattice with specific compositions close to that of the nucleated IMCs. Although various grain refinement techniques for alloys have been tried in refining Fe-rich IMCs, little progress has been reported in the literature so far.

Refinement of Fe-rich IMCs during the solidification process by enhancing heterogeneous nucleation is an important approach to deal with the Fe problem in recycled Al alloys. However, developing an effective way to enhance the heterogeneous nucleation of Fe-rich IMCs requires deep understanding on the nucleation and formation of the Fe intermetallic compounds. Our research group has focused on dealing with the problems in the past decade and new understandings on heterogeneous nucleation of Fe-rich IMCs were achieved^30–38^. Specifically, nucleation undercooling of FIMCs was measured and the interfaces between FIMCs and nucleation substrates were investigated. It has been found that due to the requirement of both the multi-component compositional and structural templating on the nucleation substrate surface, heterogeneous nucleation of Fe-rich IMCs requires an extra-large nucleation undercooling^30^, indicating the nucleation difficulty^31–33^. Following pre-nucleation, at a temperature below the liquidus, heterogeneous nucleation of an alloy occurs by structural templating and proceeds by a layer-by-layer growth mechanism^39,40^. Therefore, heterogeneous nucleation can be enhanced by reducing the lattice misfit. Recent report showed that the mismatching can be manipulated by interfacial segregation at the solid/liquid interface at the prenucleation stage, which either promotes or impedes heterogeneous nucleation^31^.

Due to the requirement of multiple constitute alloy elements for heterogeneous nucleation of Fe-rich IMCs, a composition templating hypothesis for heterogeneous nucleation of intermetallic compounds was proposed in this work, with specially designed experiments being conducted to validate the hypothesis. In the experiments, AlB_2_/TiB_2_ hybrid particles were synthesized through chemical reaction between Ti and B in an aluminium melt with excess B. Fe segregation on the boride/liquid interface was made by adding Al–Fe master alloy and isothermally holding for sufficient period of time at high temperature. Interfacial segregation of Fe and other impurities such as Si at the boride/liquid interface were carefully examined using transmission electron microscopy (TEM) and high resolution scanning transmission electron microscopy (STEM). The refinement of the primary α-Al_15_(Fe, Mn)_3_Si_2_ phase in an Al-5 Mg-2Si-1Fe-0.7Mn alloy was assessed by adding the resultant master alloy which contained Fe and Si modified boride particles. Variations in atomic arrangements at the interface due to the interfacial segregation were investigated and simulated in order to understand the composition templating effects on heterogeneous nucleation of Fe-rich IMCs.

## Experimental method

### Synthesis of AlB_2_ in Al-Ti-B Alloys

Borides particles were in-situ synthesized in an Al-2.81Ti-1.78B master alloy where there was 0.52 wt.% excess boron. The master alloy was prepared by melting commercial purity aluminium (CP-Al, > 99.86 wt. % Al) at 800 °C, followed by addition of Al-10Ti and Al-5B master alloys. The actual chemical compositions and impurities of the starting materials used in this work are given in Table 1. The alloy melts were fully homogenised by stirring until the synthesis reaction was completed. The process for synthesis reaction with mixing continued for 4 h. A certain amount of Al-38 wt.% Fe master alloy was then added to the prepared Al-2.8Ti-1.8B melt, aiming to achieve interfacial segregation of Fe on the surface of AlB_2_ particles. The Al-Ti-B-Fe melt was isothermally hold for further 4 h at 800 °C to ensure the interfacial segregation of Fe. During the holding, the Al-2.8Ti-1.8B-1.0Fe melt was manually stirred every half an hour and finally cast in a steel mould, producing sheet samples 1–5 mm thick. The composition of the resulted Al-2.8Ti-1.8B-1.0Fe sheets was measured by Inductively Coupled Plasma (ICP) analysis, as given in Table 1. Si, Mn and Ni are from the impurities in the master alloys used in the alloy preparation.

**Table 1:** Compositions of the materials used on this work.

Alloy	Wt.%									Supplier
	Al	Si	Mg	Fe	Mn	Ti	Ni	Zn	B	
CP Al	Balance	< 0.04	–	< 0.06	–	–	–	–	–	Norton
Al-10Ti	Balance	0.12	0.02	0.35	–	10.7	–	–	–	Affilips
Al-5B	Balance	0.09	–	0.17	–	–	–	–	5.44	Aleastur
Al-38Fe	60–70	0.05	–	38	–	–	–	–	–	Avon
Al-2.8Ti-1.8B-1.0Fe		0.19	–	1.02	0.02	2.81	0.01	–	1.78	This work

### Characterization

Morphology and size distribution of the synthesized borides particles in the Al-2.8Ti-1.8B-1.0Fe alloy were examined by scanning electron microscopy (SEM) using a Zeiss Supera 35 instrument, operated at an accelerating voltage of 20 kV. The EBSD measurements were made on a Zeiss Cross beam 340 FIB-SEM. The scanning step size was 0.1–0.2 μm. Specimens for TEM and STEM examinations were prepared by slicing from the Al-2.8Ti-1.8B-1.0Fe master alloy sheet containing borides particles, with the 3 mm diameter discs being ground to a thickness of about 50 µm before further thinning by Ar ion beam milling using a Gatan precision ion polishing system (PIPS) under a voltage of 1.0–5.0 kV and an incident beam angle of 3–5°. 3D morphology of the borides was observed in a sample deep-etched by a 15 vol.% HCl and H_2_O solution. The master alloy sample was etched in the solution for 60 s and then immediately washed in the running water for 30 s followed ethanol bath for 5 min.

Interface between Al and borides was examined by high resolution TEM (HRTEM) to reveal interfacial segregation. TEM examination was performed on a JEOL 2100F microscope equipped with energy dispersive x-ray analysis (EDX) operated at an accelerating voltage of 200 kV. Atomic resolution STEM with Z-contrast high-angle annular dark filed (HAADF) imaging was carried out on an aberration (Cs)-corrected FEI Titan 80–200 instrument equipped with Super-X energy dispersive x-ray spectroscopy (Super-X EDS) system, operated at an accelerating voltage of 200 kV.

The HAADF images of the interfacial segregation of Fe at the Al/boride interface were further assessed by TEM/STEM simulations using the Quantitative TEM/STEM Simulations (QSTEM) software^41^. The parameters used for the simulation were Spherical Abeer. C3 = 0 mm, Cc = 1 mm, Defu-schezer convergent angle = 30 mrad, ADF detector with an inner (outter) collection angle of (48) 196 mrad.

### Refinement test

The nucleation potency of the AlB_2_ particles as the substrates for nucleation of Fe-containing intermetallic was expected to change before and after the interfacial segregation of Fe. Effect of the AlB_2_ particles in Al-2.8Ti-1.8B-1.0Fe alloy on the size of the primary Fe-containing intermetallic was then investigated in an Al-5 Mg-2Si-1.0Mn-1.2Fe alloy, which was prepared at 750 °C with CP-Al, CP-Mg, Al-20Mn, Al-50Si, and Al-38Fe master alloys. Pure Al and the corresponding master alloys except CP-Mg were heated in an electric resistance furnace up to 750 °C. The alloy melt was held and stirred to ensure the dissolution and uniformity of the alloying elements. CP-Mg was then added to the prepared alloy melt with carefully stirring. After fully molten, the alloy melt was isothermally held at 750 °C for further 30 min. Before each casting, the melt was thoroughly stirred to ensure the homogeneity and the slag on the surface of the melt was removed. The melt was cast into a “mushroom” steel mould. The mushroom sample was manually ground for the composition test which was carried using foundry master spark chemical analyses. The prepared alloy melt was separated into two equal amounts for the casting without and with 1000 ppm (0.1%) Al-2.8Ti-1.8B-1.0Fe master alloy containing modified AlB_2_ particles respectively. The melt was casted into the pre-heated TP-1 mould (380 °C) which was immediately cooled by a water spray with a controlled water flow rate of 3.8 L/min^42^.The melt was casted within 20 min after the addition of the master alloy. The rest of alloy melt with and without the master alloy addition was further cooled inside the furnace at a low cooling rate of about 0.01 K/s. The primary α-Al_15_(Fe, Mn)_3_Si_2_ particles Al-5 Mg-2Si-0.1Mn-1.2Fe alloy were settled down and collected. The difference between the settled primary α-Al_15_(Fe, Mn)_3_Si_2_ particles with and without the addition of modified AlB_2_ particles were compared.

The TP-1 samples were sectioned transversely at 38 mm from the bottom of the ingot which had solidified at a cooling rate of 3.5 K/s. Metallographic specimens were made following the standard procedures. A Zeiss optical microscope fitted with the Axio Vision 4.3 image analysis system was used for the size assessment of the α-Al_15_(Fe, Mn)_3_Si_2_ particles. The mean linear intercept technique was used to quantify the size with measurement of at least 100 particles.

### Differential scanning calorimetry (DSC)

Nucleation undercooling of the primary α-Al_15_(Fe, Mn)_3_Si_2_ phase in Al-5Mn-2Si-1.0Mn-1.2Fe alloys without and with addition of 1000 ppm Al-2.8Ti-1.8B-1.0Fe master alloy was measured by DSC analysis. The DSC measurements were performed on approximately 20 mg disk specimens using NETZSCH DSC404F1 Pegasus at heating and cooling rates of 5, 20 and 45 K/min, respectively. High pure aluminum (99.999%) of approximately equal weight was used as the reference sample. At least 3 DSC runs were carried out with 3 different samples for each measurement with different parameters. The formation of Fe-IMCs is sensitive to the solidification conditions. Therefore, before DSC measurement, the samples were pre-heated to 800 °C at 20 K/min with 20 min holding and then cooled to 400 °C at 20 K/min with 30 min holding to avoid the previous sample effects (what effects?), followed by heating to 800 °C again with the designed heating rate after holding for 30 min and cooled at the same rate to room temperature. The DSC samples after each measurement were characterized by OM and SEM, with the solidified microstructure in each sample being matched the corresponding DSC peaks.

The onset temperature T_m_^onset^ of the first detectable deviation in the last heating curve is considered as the melting temperature of the alloy. The temperature of the first detectable deviation of the cooling DSC curve T_f_^end^ is assumed as the measured nucleation temperature T_n_. The temperature difference of (T_m_^onset^–T_f_^end^) or (T_L_–T_n_) is defined as the measured undercooling (nucleation undercooling). The average ∆T at each cooling rate was calculated based on the three times measurement with different samples.

## Results

### Nature of borides in Al-2.8Ti-1.8B master alloy

**Figure 1:**
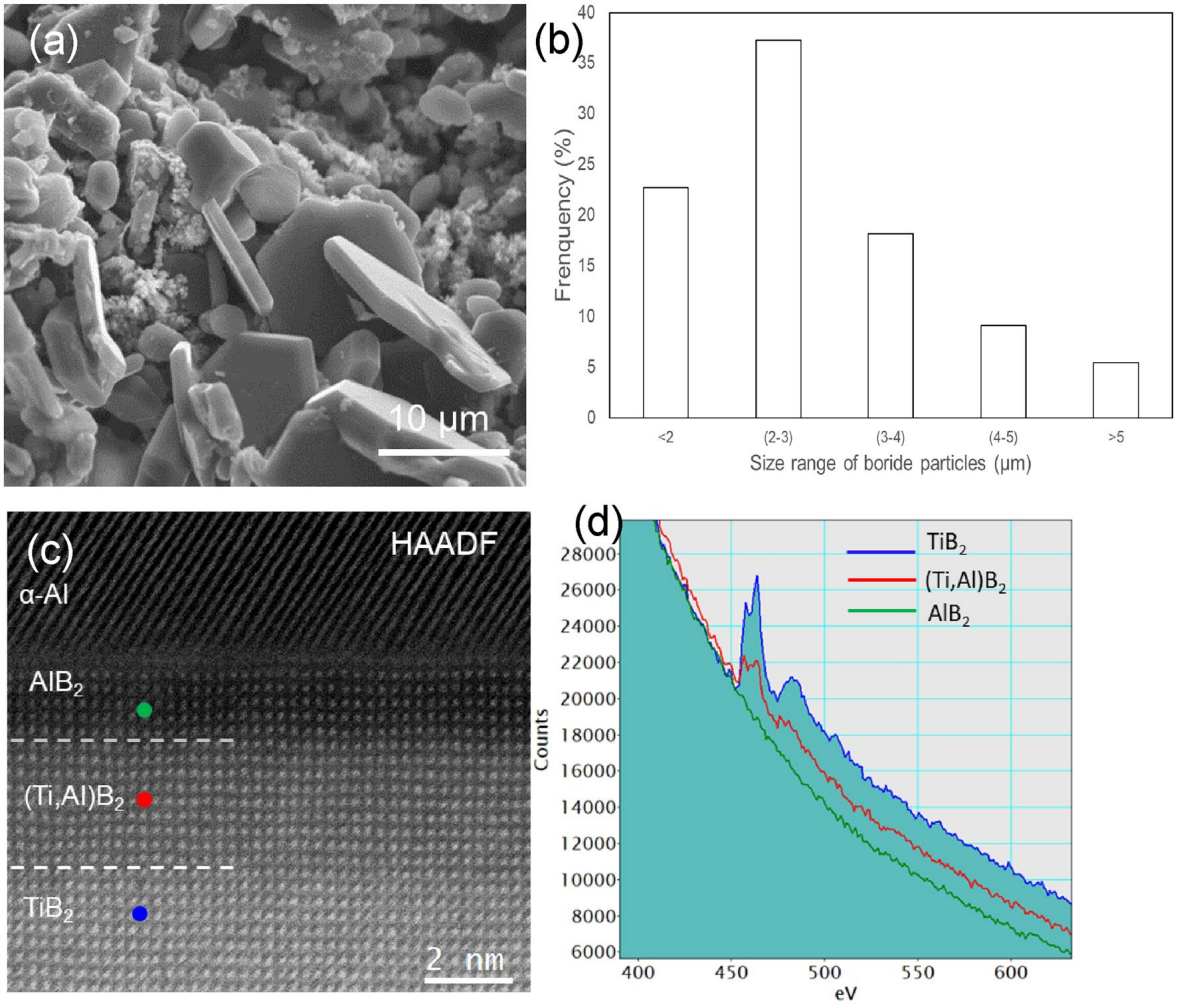
Nature of borides in Al-2.8Ti-1.8B alloy. (**a**) Scanning electron microscopy (SEM) image showing the 3-dimentional morphology of the borides in deep-etched sample, (**b**) the size distribution of boride particles, (**c**) high resolution Super STEM HAADF image across Al/(0 0 0 1)boride interface viewed along $\left[1 1 \overline{2 } 0\right]$ direction, showing the contrast variation in borides from bottom (bright) to middle (grey) and top (dark), (**d**) EELS Ti L-edge profiles variation from different positions in the boride marked in blue, red and green circles in (**b**) and suggesting the formation of AlB_2_/(Al,Ti)B_2_/TiB_2_ hybrid crystal structures of borides.

Boride particles were in-situ synthesised in an Al-2.8Ti-1.8B master alloy (with 0.52 wt.% excess B). Figure 1a shows that the borides in the master alloy have the typical hexagonal morphology. The size distribution of the boride particles was shown in Fig. 1b, with the average size being 2.96 ± 1.8 µm. The high-resolution STEM HAADF image in Fig. 1c shows the interface across Al/(0 0 0 1) AlB_2_-TiB_2_ boride viewed along $\left[1 1 \overline{2 } 0\right]$ direction of the boride, revealing the composition difference (brightness contrast) in the boride particle from bottom (bright) to middle (grey) and top (dark). The EELS spectra in Fig. 1d acquired from the three different positions (marked with blue, red and green circles in Fig. 1c) of the boride particle shows that the Ti peak inside of the particle (blue) is highest and gradually reduced to zero when near the Al/AlB_2_ interface. With a few atomic layers in the boride particle from the Al/TiB_2_ interface (green), no Ti signal was detected. The thickness of these AlB_2_ layers in different borides particles varied from a few to a few tens of atomic layers. This character fits most of the terminated planes of the borides in this study, indicating that the synthesized borides in the Al-2.8Ti-1.8B alloy are hybrid AlB_2_/(Al,Ti)B_2_/TiB_2_ where AlB_2_ covered the whole surface of the particle. Heterogeneous nucleation on the borides would depend on only the atomic arrangement on the surface of the substrate. Therefore, in this study, these synthesised borides were named AlB_2_ instead of hybrid AlB_2_/(Al, Ti)B_2_/TiB_2_.

### Segregation of Fe et al./AlB_2_ interface

**Figure 2:**
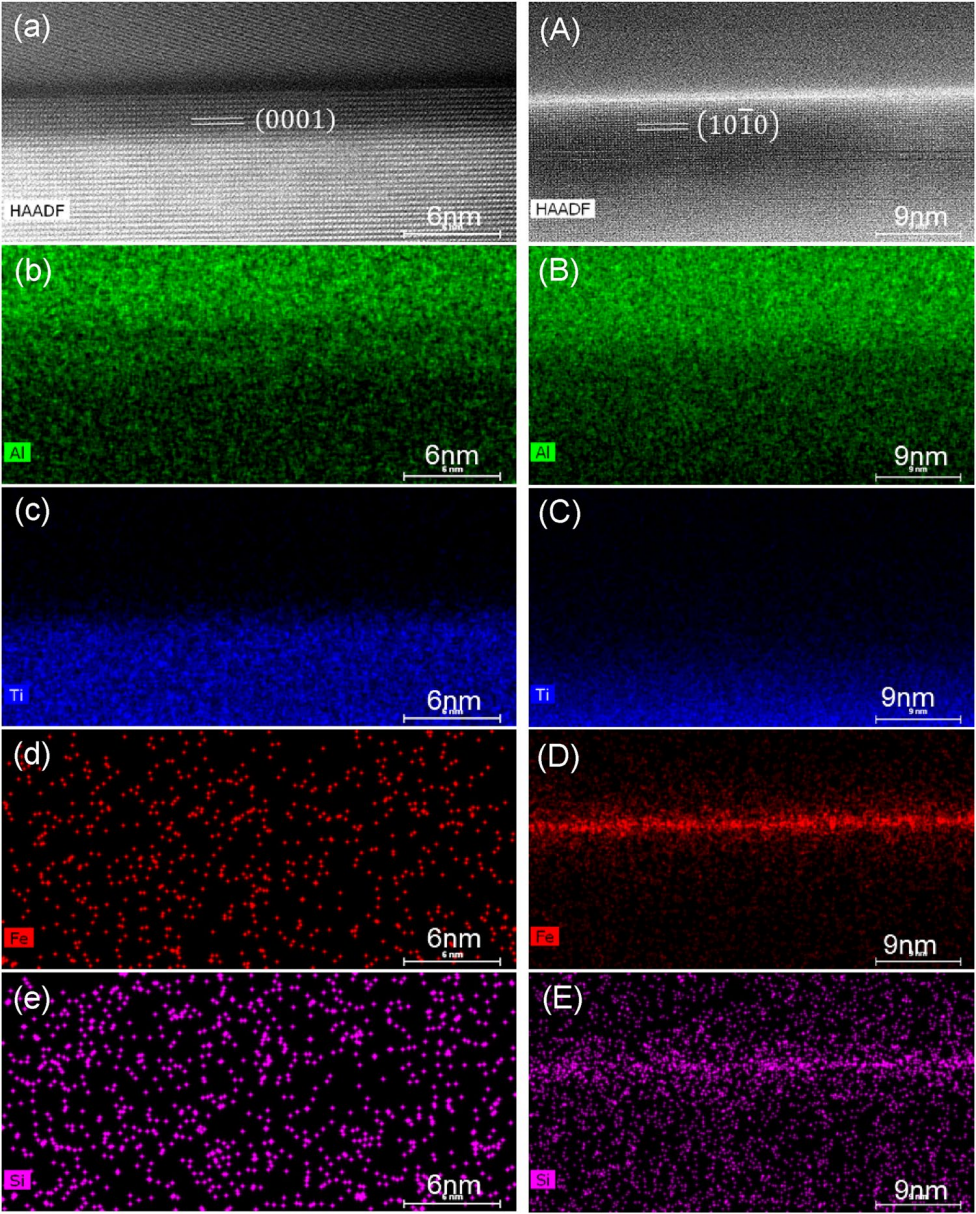
Identification of elemental segregation et al./AlB_2_ interface in Al-2.8Ti-1.8B-1.0Fe alloy. (**a**) and (A) High resolution STEM HAADF images across the (**a**) Al/(0 0 0 1)AlB_2_ and (A) Al/$\left(1 0 \overline{1 } 0\right)$ AlB_2_ interface viewed along $\left[1 1 \overline{2 } 0\right]$ AlB_2_ direction, (**b**–**e**) Super-X EDS elemental mapping of Al/(0 0 0 1)AlB_2_ interface: (**b**) Al (lime), (**c**) Ti (blue), (**d**) Fe (red), (**e**) Si (pink); and (B-E) Super-X EDS elemental mapping of Al/$\left(1 0 \overline{1 } 0\right)$ AlB_2_ interface: (B) Al (lime), (C) Ti (blue), (D) Fe (red), (E) Si (pink); showing that no Fe and Si segregation on Al/(0 0 0 1)AlB_2_, but obvious Fe and Si segregation on $\left(1 0\overline{ 1 } 0\right)$ planes of AlB_2_.

With the hybrid borides being synthesized, 1 wt.% Fe was added to the Al-Ti-B master alloy melt, aiming to achieve Fe segregation at the Al/AlB_2_ interface. The AlB_2_ particles with hexagonal crystal structure were normally terminated with their (0 0 0 1) and $\left(1 0 \overline{1 }0\right)$ planes. STEM HADDF images and super-X EDS elemental mapping in Fig. 2 show the compositional profiles of alloying elements at the Al/(0 0 0 1)AlB_2_ (Fig. 2a–e) and Al/$\left(1 0 \overline{1 }0\right)$ AlB_2_ (Fig. 2A–E) interfaces. The EDS elemental mappings of Al, Ti, Fe and Si on these two interfaces are displayed in Fig. 2b–e and Fig. 2B–E, respectively. No obvious interfacial segregation of any element was detected at the Al/(0 0 0 1)AlB_2_ interface. However, Fe as the added alloying element and Si as the major impurity in Al-2.8Ti-1.8B-1.0Fe master alloy are seen to obviously segregate at the Al/$\left(1 0 \overline{1 }0\right)$ AlB_2_ interface.

**Figure 3:**
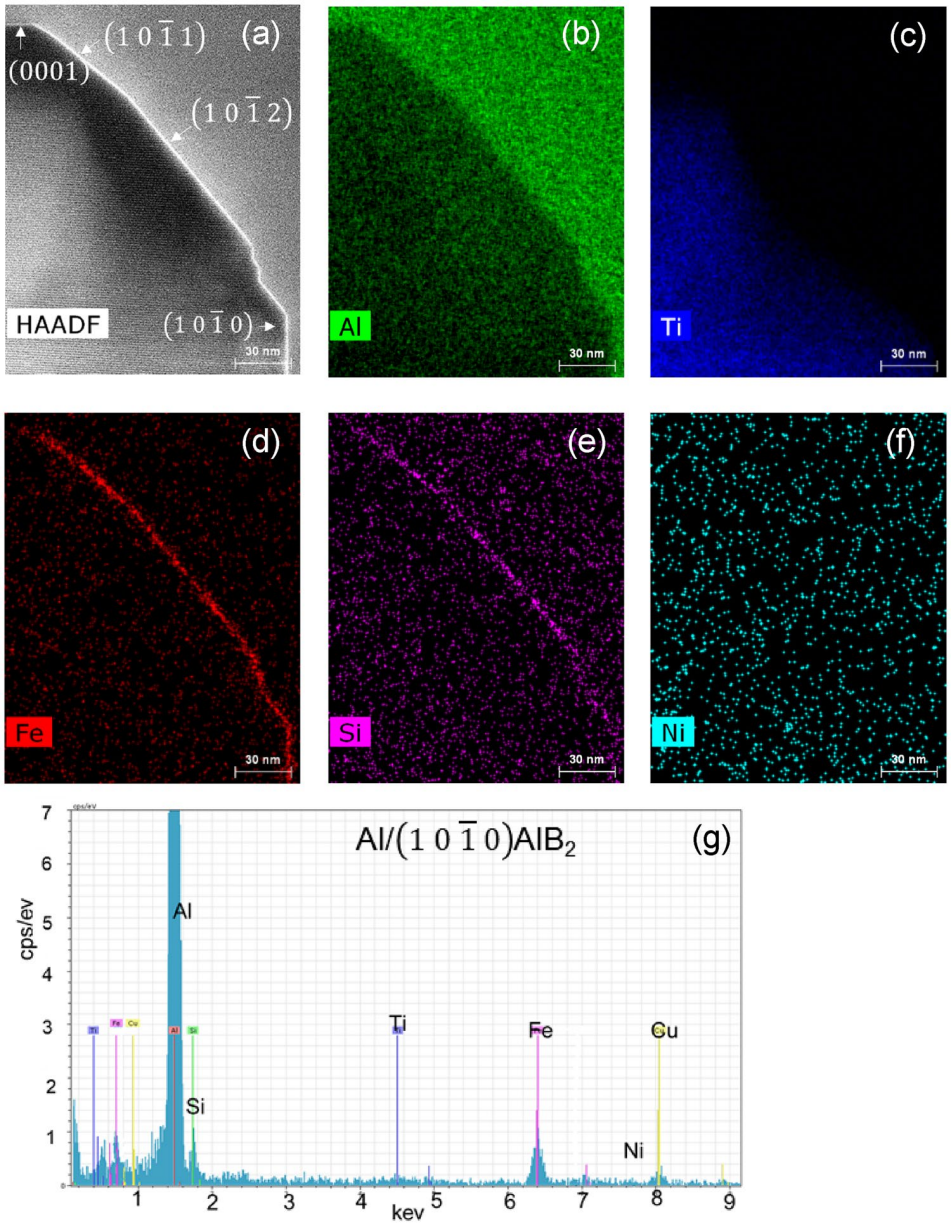
Identification of elemental segregation et al./AlB_2_ interface in Al-2.8Ti-1.8B-1Fe alloy. (**a**) High resolution STEM HAADF image across the Al/AlB_2_ interface viewed along $\left[1 1 \overline{2 } 0\right]$ AlB_2_ direction, and (**b**–**f**) Super-X EDS elemental mapping of (**b**) Al (lime), (**c)** Ti (blue), (**d**) Fe (red), (**e**) Si (pink) and (**f**) Ni, showing Fe and Si segregation on $\left(1 0 \overline{1 } 0\right)$, $\left(1 0 \overline{1 } 1\right)$, and $\left(1 0 \overline{1 } 2\right)$ planes of AlB_2_; (**g**) EDS spectrum acquired from the surface of AlB_2_ particles on $\left(10\overline{1 }0\right)$ plane.

Although the AlB_2_ particles are mainly terminated with (0 0 0 1) and $\left(1 0 \overline{1 } 0\right)$ planes, $\left(1 0 \overline{1 } 1\right)$ and $\left(1 0 \overline{ 1 } 2\right)$ terminated planes were also observed occasionally as the transition planes from basal to prismatic. Figure 3 shows the STEM HAADF image of an AlB_2_ particle and the corresponding Super-X energy dispersive x-ray spectroscopy (Super-X EDS) elemental mappings of Al, Ti, Fe, Si and Ni, verifying Fe segregation on the $\left(1 0\overline{ 1 } 0\right)$, $\left(1 0\overline{ 1 } 1\right)$ and $\left(1 0 \overline{1 } 2\right)$ surfaces, but not on (0 0 0 1) of the AlB_2_. Fe segregation on the AlB_2_ surface is characterised by the brighter contrast of the interfacial segregation layer than the Al layer of the AlB_2_ particle. As given in Table 1, the major impurities in the master alloys used in the work are Si (~ 0.19%), Mn (0.02%) and Ni (0.01%). The EDS mapping in Fig. 3e shows Si segregation at the Al/$\left(1 0 \overline{1 } 0\right)$ AlB_2_, Al/$\left(1 0 \overline{1 } 1\right)$ AlB_2_ and Al/$\left(1 0 \overline{1 }2\right)$ AlB_2_ interfaces. Si is the impurities from the master alloys for the synthesized of Al-2.8Ti-1.8B-1.0Fe alloy. However, neither Ni nor Mn was detected on any surface of the AlB_2_ particles.

Figure 3g presents the STEM-EELS EDS spectrum acquired from the interface region of Al/$\left(1 0 \overline{1 } 0\right)$ AlB_2_. Four elements Al, Si, Fe and Cu were detected, with the major peak being from the Al matrix and also probably from the segregation layer at the Al/$\left(1 0 \overline{1 } 0\right)$ AlB_2_ interface. Obviously, the Cu peak is from the specimen holder (the washer and sample cramp are made of Cu).

**Figure 4:**
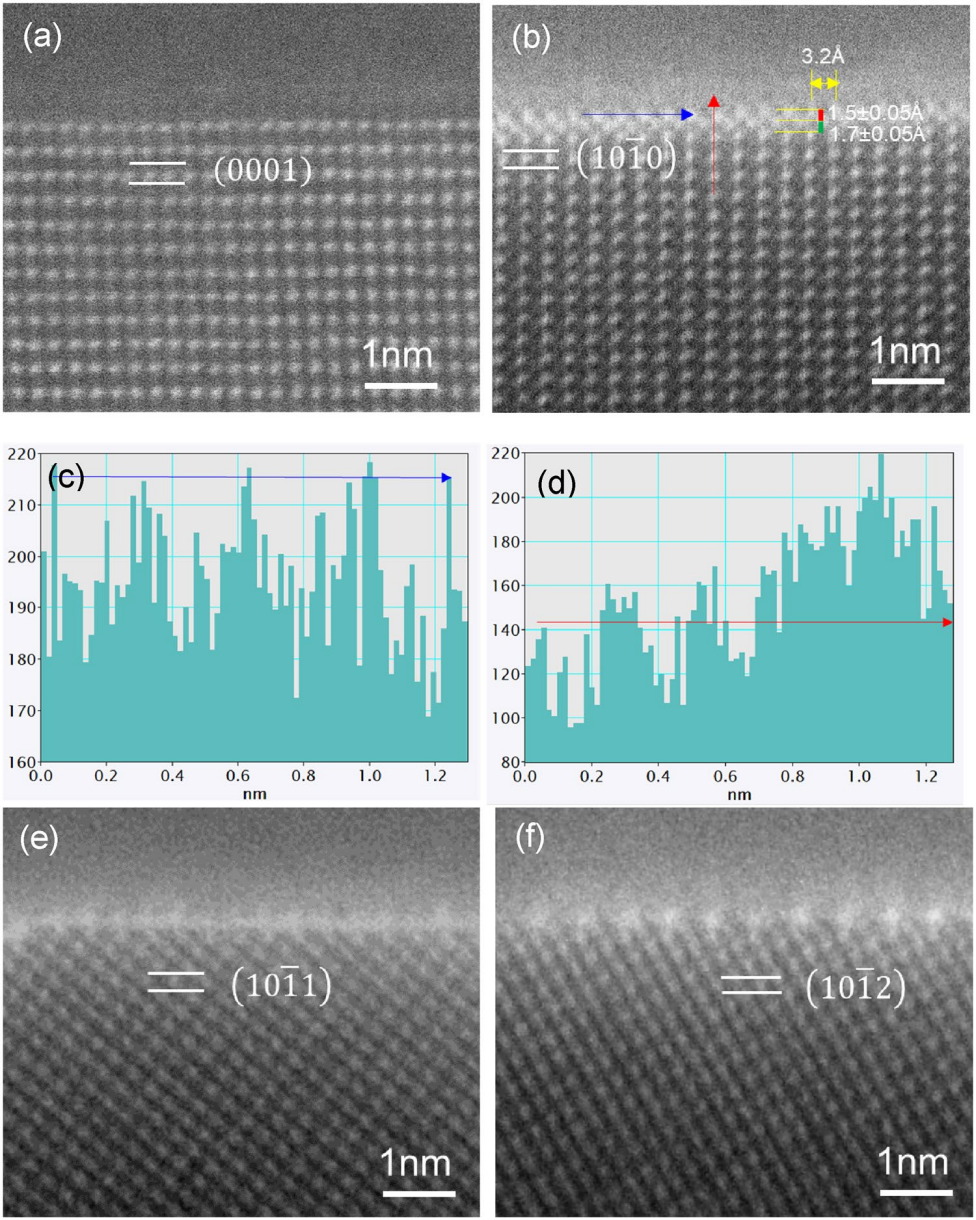
Atomic arrangement of segregation layers et al./AlB_2_ interface viewed along $\left[1 1 \overline{2} 0\right]$ AlB_2_ direction. STEM HAADF images of, (**a**) Al/$\left(0 0 0 1\right)$ AlB_2_ interface, (**b**) Al/$\left(1 0 \overline{1 } 0\right)$ AlB_2_ interface, and the EELS profile variation at (**c**) the Fe segregation layer (blue arrow in (**b**)) and from (**d**) AlB_2_ to the segregation layers (red arrow in (**b**)), (**e**) Al/$\left(1 0 \overline{1 } 1\right)$ AlB_2_ interface and (**f**) Al/$\left(1 0 \overline{1 } 2\right)$ AlB_2_ interface.

Figure 4a shows the Al/(0 0 0 1)AlB_2_ interface where no atomic arrangement is different from that of the AlB_2_, apparently indicating no elemental segregation. However, extra atomic layers with a zig-zag fashion are clearly seen at the Al/$\left(1 0\overline{ 1 } 0\right)$ AlB_2_ interface shown in Fig. 4b. The segregation layers are slightly brighter than Al layers of AlB_2_ (Fig. 4d) with no variation in brightness between the atomic columns of the segregation layers (Fig. 4c). The planar spacings from the top Al layer of the AlB_2_ to the first layer of the segregation layers, and from the first layer to the second layer were measured as 1.7 ± 0.05 Å and 1.5 ± 0.05 Å, respectively, compared to 2.6 Å, the d-spacing of $\left(1 0 \overline{1 } 0\right)$ AlB_2_ planes.

As shown in the STEM HAADF images in Fig. 4e and f, there are brighter atomic columns at both the Al/$\left(1 0\overline{ 1 } 1\right)$ AlB_2_ and Al/$\left(1 0 \overline{1 } 2\right)$ AlB_2_ interfaces, also revealing atomic segregation at the interfaces. The segregation monolayers at both the interfaces are found to exhibit periodic bright and dark variation.

### Refinement of Fe-rich IMCs

**Figure 5:**
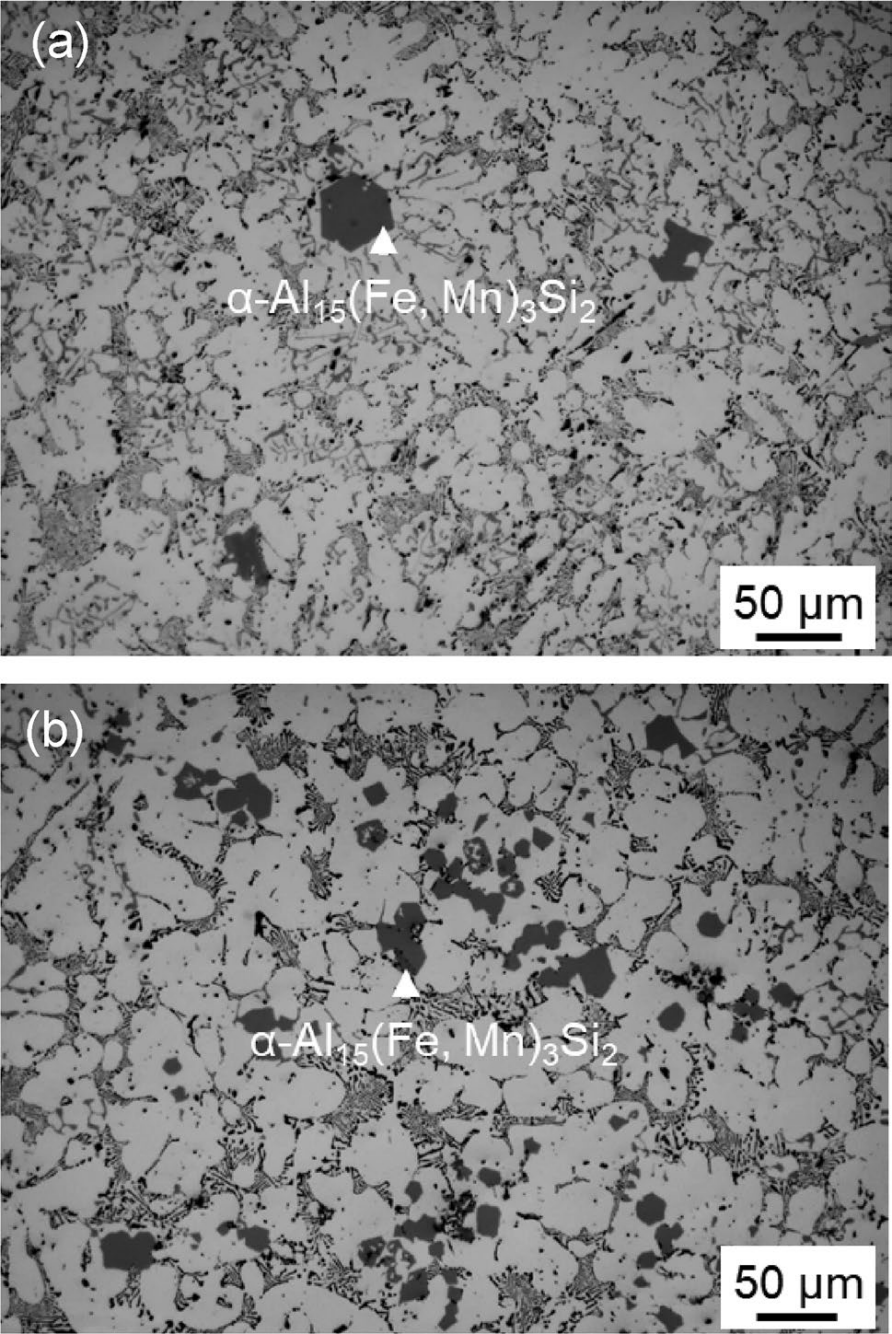
Optical microstructure of Al-5 Mg-2Si-1.0Mn-1.2Fe alloy cast at 700 ℃ with cooling rate of 3.5 K/s: (**a**) before and (**b**) after added 1000 ppm Al-2.8Ti-1.78B-1Fe master alloy containing the AlB_2_ particles with interfacial segregation of Fe.

By addition of the Al-2.8Ti-1.8B-1.0Fe master alloy, the synthesized AlB_2_ particles covered by Fe segregation layers on their surfaces were introduced into an Al-5 Mg-2Si-1.0Mn-1.2Fe alloy melt, aiming to refine the primary Fe-IMCs. The refinement efficiency was assessed at a cooling rate of 3.5 K/s and with an addition rate of 1000 ppm of the master alloy. Figure 5 shows the general as-cast microstructure of the Al-5 Mg-2Si-1.0Mn-1.2Fe alloy with and without the addition of the borides, where the primary α-Al_15_(Fe, Mn)_3_Si_2_ particles appear dark grey with a compact morphology. Experimental measurement revealed that, by the addition of the AlB_2_ particles, the size of the primary intermetallic compounds was halved, with the average size decreasing from 38.7 ± 6.8 μm to 19.2 ± 5.6 μm. Corresponding to the decrease in size, the number density of the primary intermetallic phase increased significantly.

**Figure 6:**
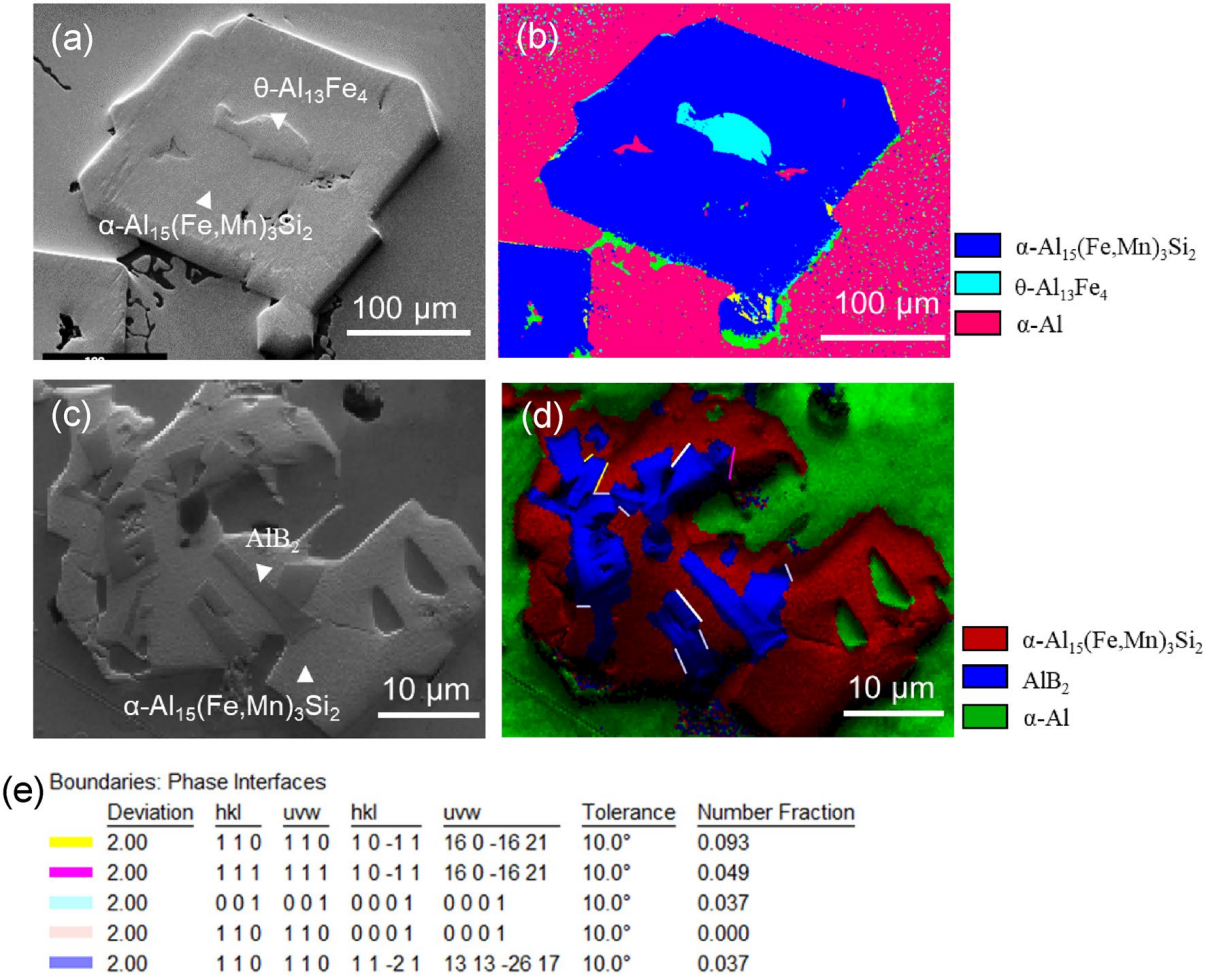
Different types of particles were engulfed in the settled primary α-Al_15_(Fe, Mn)_3_Si_2_ in Al-5 Mg-2Si-1.0Mn-1.2Fe alloy solidified at 0.01 K/s without (**a**,**b**) and with grain refiner addition (**c**–**e**). (**a**) SEM image showing a different particle with irregular morphology was engulfed inside of the primary α-Al_15_(Fe, Mn)_3_Si_2_ which was identified as θ-Al_13_Fe_4_ in (**b**) the phase image of the Electron Backscatter Diffraction (EBSD) mapping; (**c**) SEM image showing multiple particles with rectangle shape were embedded in the primary α-Al_15_(Fe, Mn)_3_Si_2_ which was identified as AlB_2_ in (**d**) the phase image of the EBSD mapping, and (**e**) the misorientation of the engulfed AlB_2_ particles from EBSD mapping of (**d**).

To understand the effects of modified AlB_2_ particles on heterogeneous nucleation of the primary α-Al_15_(Fe, Mn)_3_Si_2_ particles, the alloy was also solidified at a very slow cooling rate of ~ 0.01 K/s by cooling inside the furnace and the intermetallic particles were collected at the bottom of the crucible by sediment. Figure 6a shows the collected primary α-Al_15_(Fe, Mn)_3_Si_2_ particles in Al-5 Mg-2Si-1.0Mn-1.2Fe alloy without addition of the master alloy, where there is apparently a different phase with irregular shape inside each of the α-Al_15_(Fe, Mn)_3_Si_2_ particles. The phase inside has been identified as θ-Al_13_Fe_4_ by means of electron backscatter diffraction (EBSD) mapping as shown in Fig. 6b. The details of the formation mechanism of α-Al_15_(Fe, Mn)_3_Si_2_ particles in Al-5 Mg-2Si-1.0Mn-1.2Fe alloy without addition of grain refiner have been investigated in our previous publication^32^. It was found that the α-Al_15_(Fe, Mn)_3_Si_2_ is actually formed by phase transformation from the θ-Al_13_Fe_4_ that previously had nucleated on native MgAl_2_O_4_ particles as a non-equilibrium intermetallic. This indicates that heterogeneous nucleation of the primary α-Al_15_(Fe, Mn)_3_Si_2_ is more difficult than θ-Al_13_Fe_4_ in Al–Mg-Si-Mn-Fe alloys.

It is interesting to note that, as shown in Fig. 6c and d, no primary θ-Al_13_Fe_4_ particles were observed inside the primary α-Al_15_(Fe, Mn)_3_Si_2_ particles when Al-2.8Ti-1.8B-1.0Fe master alloy containing the AlB_2_ particles with Fe and Si interfacial segregation was added. Instead, AlB_2_ particles were frequently seen in the primary α-Al_15_(Fe, Mn)_3_Si_2_ particles. This indicates strongly that heterogeneous nucleation of α-Al_15_(Fe, Mn)_3_Si_2_ particles have taken place on the surface of the modified AlB_2_ particles. Figure 6c,d also show that there are multiple AlB_2_ particles imbedded in one α-Al_15_(Fe, Mn)_3_Si_2_ particle. The misorientation of these AlB_2_ particles shown in the EBSD mapping (Fig. 6d) was analysed and displayed in Fig. 6e. The number fractions of different orientation AlB_2_ particles imbedded in the α-Al_15_(Fe, Mn)_3_Si_2_ particle can be seen from Fig. 6e. These AlB_2_ particles are possibly from the agglomeration and trapped in the α-Al_15_(Fe, Mn)_3_Si_2_ particle once nucleation happened during the solidification progress. Principally, only one AlB_2_ particle in an α-Al_15_(Fe, Mn)_3_Si_2_ particle serves as the nucleation substrate. The direct evidence of heterogeneous nucleation of α-Al_15_(Fe, Mn)_3_Si_2_ particles on the modified AlB_2_ particles is required.

### Heterogeneous nucleation of FIMCs on AlB_2_(Fe)

**Figure 7:**
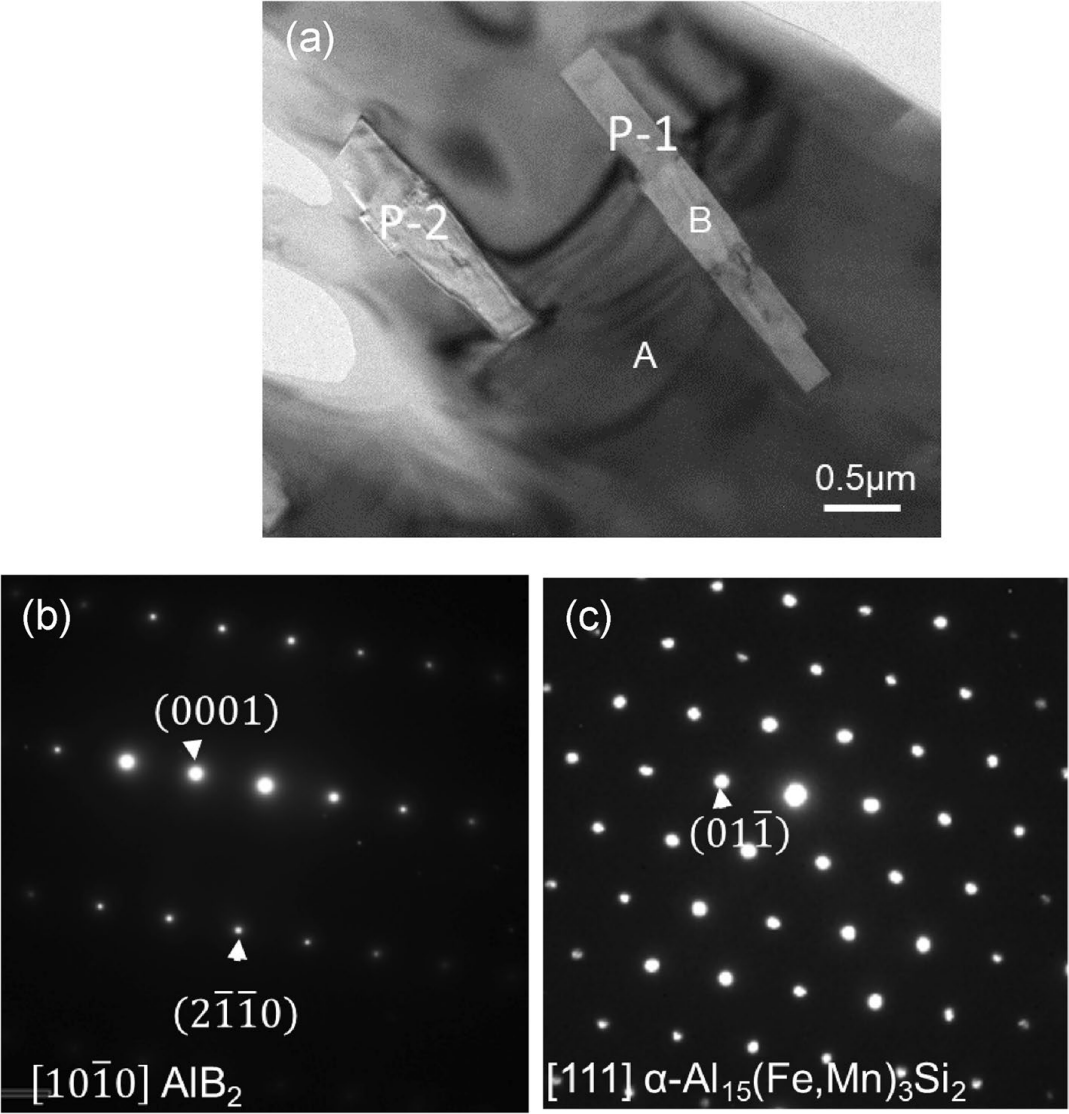
(**a**) TEM bright field image showing the Fe-modified AlB_2_ particles (marked with B) embedded in α-Al_15_(Fe,Mn)_3_Si_2_ intermetallic compound (marked with A), (**b**) and (**c**) are indexed selected area electron diffraction patterns taken from the boride particle and the intermetallic phase with $\left[1 0 \overline{1 } 0\right]$ and [1 1 1] zone direction, respectively.

**Figure 8:**
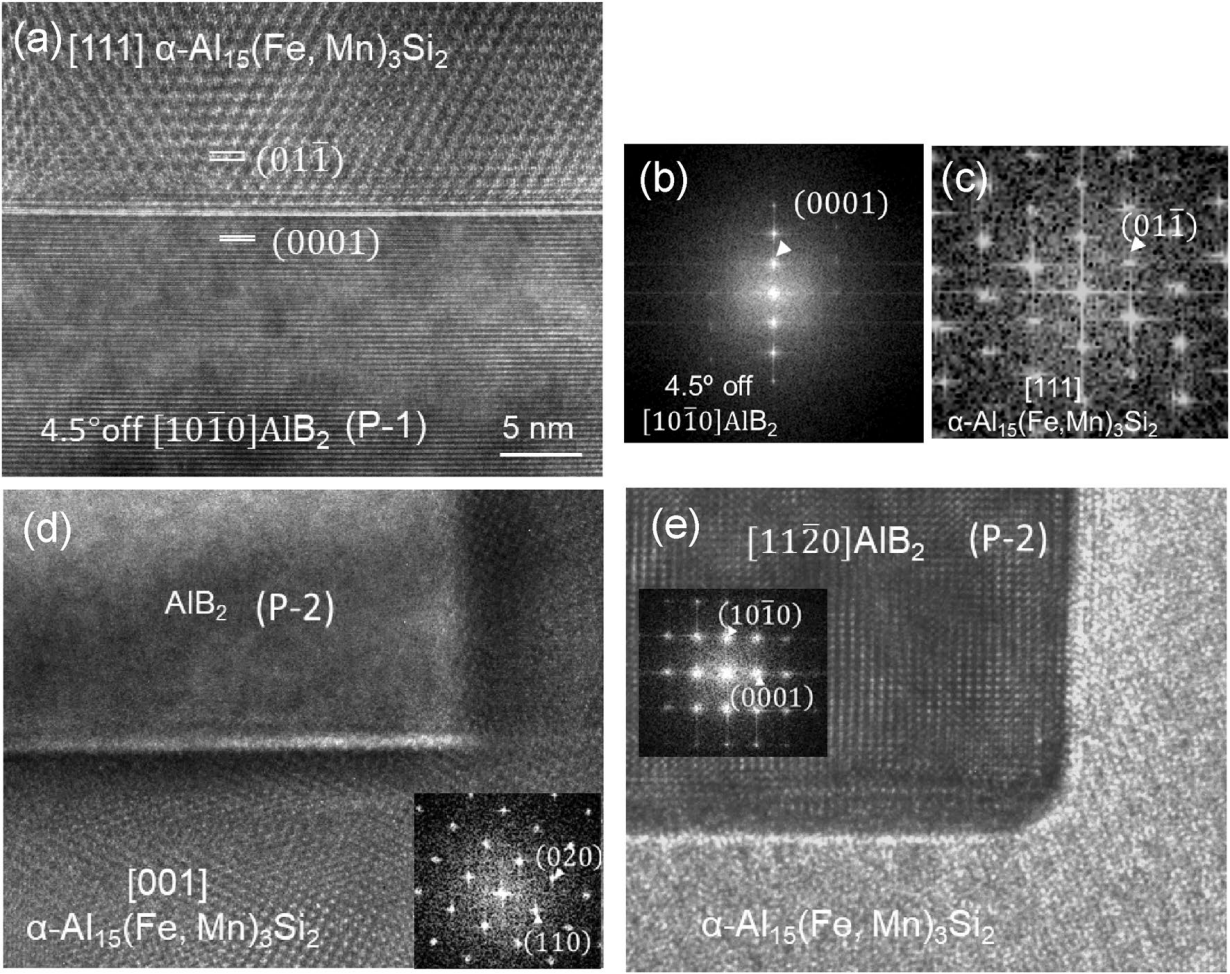
(**a**) High resolution TEM image showing the interface between AlB_2_ particle (P-1 in Fig. 7a) and α-Al_15_(Fe,Mn)_3_Si_2_ phase with AlB_2_ and α-Al_15_(Fe,Mn)_3_Si_2_ being viewed along $\left[10\overline{1 }0\right]$ (4.5° deviation) and [111], respectively; (**b**) and (**c**) are indexed Fast Fourier transformation (FFT) patterns for AlB_2_ and α-Al_15_(Fe,Mn)_3_Si_2_, respectively, (**d**) and (**e**) are the HRTEM images including the indexed FFT of the interface between particle 2 (P-2 in Fig. 7a) when viewed along (**d**) the [001] of α-Al_15_(Fe,Mn)_3_Si_2_ and (**e**) the $\left[11\overline{2 }0\right]$ AlB_2_, indicating that the P-2 doesn’t have orientation relationship with α-Al_15_(Fe,Mn)_3_Si_2_ and not serves the nucleation substrate also.

TEM image in Fig. 7a shows two AlB_2_ particles (P-1 and P-2) inside an α-Al_15_(Fe, Mn)_3_Si_2_ particle (marked with A). Figures 7b,c are the indexed selected area electron diffraction (SAED) patterns taken from P-1 particle in $\left[1 0 \overline{1 } 0\right]$ direction and the α-Al_15_(Fe, Mn)_3_Si_2_ in [1 1 1] direction, respectively. High resolution TEM (HRTEM) image in Fig. 8a shows the interface between the P-1 AlB_2_ particle and α-Al_15_(Fe, Mn)_3_Si_2_ particle, where this boride is viewed along a direction 4.5° deviated from the $\left[1 0 \overline{1 } 0\right]$ zone and the Al_15_(Fe, Mn)_3_Si_2_ is perfectly in its [1 1 1] zone. The corresponding fast Fourier transformation (FFT) patterns shown in Fig. 8b,c reveal the specific OR1 below:

OR1 $$\begin{gathered} \left( {0\,1\,\overline{1}} \right)\alpha - {\text{Al}}_{{{15}}} \left( {{\text{Fe}},{\text{ Mn}}} \right)_{{3}} {\text{Si}}_{{2}} // \, \left( {0 \, 0 \, 0{ 1}} \right){\text{ AlB}}_{{2}} ,{\text{ and}} \hfill \\ \left[ {1 1 1} \right]\alpha - {\text{Al}}_{{{15}}} \left( {{\text{Fe}},{\text{ Mn}}} \right)_{{3}} {\text{Si}}_{{2}} \sim { 4}.{5}^{ \circ } \,\left[ {10\,\overline{1}\,0} \right]\,{\text{AlB}}_{{2}} . \hfill \\ \end{gathered}$$

However, not all of the AlB_2_ particles inside the intermetallic have a defined OR with the intermetallic particle. No such orientation relationship between the other AlB_2_ particles P-2 and the α-Al_15_(Fe, Mn)_3_Si_2_ phase can be observed, as shown in Fig. 8d,e. This indicates that some of the AlB_2_, the P-1 particle for instance, had acted as the substrate for heterogeneous nucleation of the α-Al_15_(Fe, Mn)_3_Si_2_ phase and the other AlB_2_ particles P-2 were simply engulfed in the same intermetallic particle during growth.

The deviation by 4.5° between [1 1 1] α-Al_15_(Fe, Mn)_3_Si_2_ and $\left[1 0 \overline{1 } 0\right]$ AlB_2_ indicates that the nucleated α-Al_15_(Fe, Mn)_3_Si_2_ phase on the (0 0 0 1) AlB_2_ surface is twisted 4.5° along $\left[1 0 \overline{1 } 0\right]$ TiB_2_ axis. It is found that when the twist angle of OR1 slightly increases from 4.5° to 5.3°, the orientation relationship is equivalent to a new OR below:

OR2 $$\begin{gathered} \left( {1\,0\,\overline{1}\,0} \right)\,{\text{AlB}}_{{2}} // \, \left( {0 \, 0{ 1}} \right)\alpha - {\text{Al}}_{{{15}}} \left( {{\text{Fe}},{\text{ Mn}}} \right)_{{3}} {\text{Si}}_{{2}} ,{\text{ and}} \hfill \\ \left[ {1\,1\,\overline{2}\,0} \right]\,{\text{AlB}}_{{2}} // \, \left[ {0{ 1 1}} \right]\alpha - {\text{Al}}_{{{15}}} \left( {{\text{Fe}},{\text{ Mn}}} \right)_{{3}} {\text{Si}}_{{2}} . \hfill \\ \end{gathered}$$

OR2 is believed to be the real nucleation OR between the AlB_2_ and α-Al_15_(Fe, Mn)_3_Si_2_. The difference by 0.8° between the experimentally recorded 4.5° and the calculated 5.3° is attributed to the experimental error. It is likely that the α-Al_15_(Fe, Mn)_3_Si_2_ had heterogeneously nucleated on the $\left(1 0 \overline{1 } 0\right)$ AlB_2_ surface, since Fe interfacial segregation occurs on the prismatic surface rather than on the basal (0 0 0 1) surface of the borides.

### Reduced nucleation undercooling of FIMCs by AlB_2_(Fe) addition

**Figure 9:**
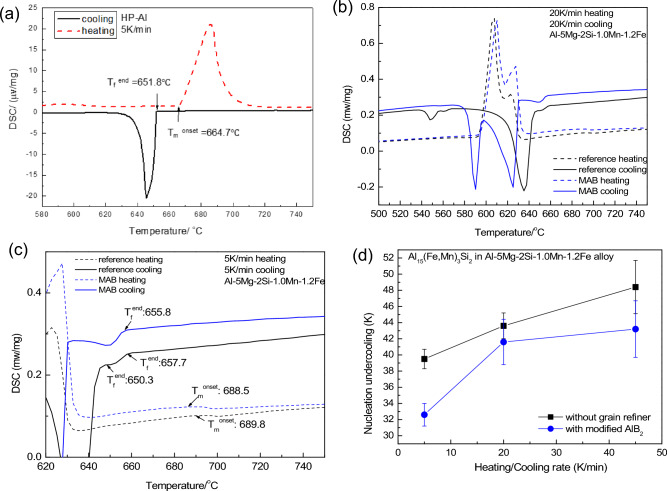
(**a**) Experimental differential scanning calorimetry (DSC) melting and freezing traces for high purity (HP) (99.999 wt.%) Al at 5 K/min, (**b**) DSC result of Al-5Mn-2Si-1.0Mn-1.2Fe alloys without and with 1000 ppm Al-2.8Ti-1.8B-1.0Fe master alloy containing modified AlB_2_ particles, heated and cooled at 5 K/min, (**c**) higher magnification of (**b**) at the melting (heating) and solidification (cooling) part of the primary phases, and (**d**) the measured nucleation undercooling (∆T) at different heating and cooling rates. *P* Primary, *BE* binary eutectic, *TE* ternary eutectic.

Nucleation undercooling of the primary α-Al_15_(Fe, Mn)_3_Si_2_ in Al-5 Mg-2Si-1.0Mn-1.2Fe alloys without and with 1000 ppm Al-2.8Ti-1.8B-1.0Fe master alloy was investigated with differential scanning calorimetry (DSC) measurement. High purity (HP) Al (99.999 wt.%) was taken as the reference for measurement of the nucleation undercooling in the DSC method. Figure 9a shows the heating and cooling traces for HP-Al at 5 K/min, where the temperature T_m_^onset^ of the first detectable deviation in the heating curve is 664.7 °C, assumed as the liquidus T_L_. The temperature of the first detectable deviation of the cooling DSC curve T_f_^end^ (651.8 °C), which is assumed as the measured nucleation temperature T_n_. The temperature difference of (T_m_^onset^–T_f_^end^) or (T_L_–T_n_) is defined as the measured undercooling (nucleation undercooling or grain initiation undercooling). In this experiment, the measured undercooling for HP-Al is 12.9 K. The DSC measurement at this parameter (both heating and cooling rate is 5 K/min) was carried out at least 3 times. And the average measured undercooling is 14.8 ± 0.3 K. This result is close to that reported undercooling of one 99.6 wt.% pure Al^43^ measured with DSC also.

The nucleation undercooling measurement of Fe-IMCs is complicated due to its sensitity to the alloy composition and solidification condition. The phase slection of FIMCs is hardly to be predicted by phase diagram calculation. Therefore, the solidification sequence for the designed studied alloys under different solidification conditions need to be investigated very carefully. The DSC peaks in each DSC measurement needs to be contraposed with the corresponding phases very well. The solidification sequence and the formation of F-IMCs in Al-5 Mg-2Si-(0.7 ~ 1.0) Mn-(1.0 ~ 1.2) Fe alloys are well investigated in our previous studies^32,34^. It normally forms the non-equilibrium primary θ-Al_13_Fe_4_ firstly which transformed into equilibrium α-Al_15_(Fe, Mn)_3_Si_2_ later during the following solidification process^32^.

The measurement was taken at differnt heating and cooling rates at 5, 20 and 45 K/min. At each experimental conditions, the DSC was runed at least three times with sample sectioned from differnt positon of the original alloys (TP-1). Figure 9b,c show an example of the DSC results of Al-5 Mg-2Si-1.0Mn-1.2Fe alloys without and with 1000 ppm Al-2.8Ti-1.8B-1.0Fe master alloy which heated and cooled at 5 K/min. Figure 9b shows that the samples have similar heating DSC traces, but have very different cooling DSC traces initiated at the primary phase formation range and the corresponding different DSC traces for eutectic formation. The DSC traces for the primary phase melting and solidification part were enlarged and shown in Fig. 9c. The cooling curve of the Al-5 Mg-2Si-1.0Mn-1.2Fe alloy without the master alloy addition shows two DSC peaks before the eutectic formation, which corresponding to θ-Al_13_Fe_4_ and α-Al_15_(Fe, Mn)_3_Si_2_ respectively. In contrast, only one peak for the primary α-Al_15_(Fe, Mn)_3_Si_2_ is seen in the cooling curve of Al-5 Mg-2Si-1.0Mn-1.2Fe alloy when the modified AlB_2_ particles was introduced. The results are in agreement with the experimental results shown in Fig. 6, which showed that without grain refiner addition, θ-Al_13_Fe_4_ phase was formed firstly prior to the formation of α-Al_15_(Fe, Mn)_3_Si_2_ phase. With the addition of modified AlB_2_ particles, the primary α-Al_15_(Fe, Mn)_3_Si_2_ phase directly nucleated on the modified AlB_2_ particles, supressing the formation of θ-Al_13_Fe_4_ phase. The difference in the formation of the primary phases affects the following eutectic reaction which have different DSC traces also. To consider the effects of modified AlB_2_ particles on the nucleation undercooling of α-Al_15_(Fe, Mn)_3_Si_2_ phase, the nucleation undercooling was calculated with the measured T_m_^onset^ and the T_f_^end^ of the primary α-Al_15_(Fe, Mn)_3_Si_2_ phase regardless of the formation of the primary θ-Al_13_Fe_4_ phase.

Figure 9d shows the measured nucleation undercooling of α-Al_15_(Fe, Mn)_3_Si_2_ phase with and without the modified AlB_2_ particle addition when heated and cooled at different rates. The results reveal following facts:

- The nucleation undercooling required for nucleation of α-Al_15_(Fe, Mn)_3_Si_2_ is a few tens of Kelvin and more than an order of magnitude higher than that for commercial pure Al^44^.
- Nucleation undercooling increases with increasing cooling rate.
- Nucleation undercooling obviously decreases with the addition of the modified AlB_2_ particles.

Heterogeneous nucleation of IMCs is obviously more difficult compared with pure metals, since it requires not only structural templating to create the crystal structure but also chemical compositions and atomic arrangement of the constitute elements within the crystal structure. The supply of the constitute atoms to the Solid/liquid interface becomes a critical factor for heterogeneous nucleation of IMCs. On the other hand, the measurement of the nucleation undercooling validates that the composition templating of constitute elements Fe and Si at the Solid/liquid interface of AlB_2_ particles does facilitate heterogeneous nucleation of the equilibrium IMC.

## Discussion

### Chemistry and structure of Fe segregation layers

STEM and EDS analysis in Figs. 2, 3, 4 have demonstrated the segregation of Fe and Si at the Al/$\left(1 0 \overline{1 } 0\right)$, Al/$\left(1 0 \overline{1 } 1\right)$ and Al/$\left(1 0 \overline{1 } 2\right)$ AlB_2_ interfaces. The elemental segregation of Fe and Si is believed to be from chemical reaction and bonding effects on the AlB_2_ particles surface. In the Al-Ti-B master alloy with excess B, the competition of interfacial segregation between different types of atoms such as Al, Fe, Si, Ni, Mn, and B on AlB_2_ surface can be assessed by the mixing enthalpies (∆H) between the alloying elements. The mixing enthalpies ∆${H}_{Al-B}^{mix}$, ∆${H}_{Al-Fe}^{mix}$, ∆${H}_{Al-Mn}^{mix}$, ∆${H}_{Al-Ni}^{mix}$ and ∆${H}_{Al-Si}^{mix}$, are 0, − 11, − 19, − 22, − 26 kJ/mol, respectively, while the ∆${H}_{B-Si}^{mix}$, ∆${H}_{B-Ni}^{mix}$, ∆${H}_{B-Fe}^{mix}$, ∆${H}_{B-Mn}^{mix}$ and are − 14, − 24, − 26, − 32 kJ/mol, respectively^45^. Fe, Si, Mn and Ni have a large negative mixing enthalpy with either Al or B, and therefore most likely tend to segregate at the interface. Indeed, segregation of Fe and Si, but not Mn and Ni, has been experimentally observed in this work, probably due to the much higher concentration of Fe and Si than Mn and Ni in the alloy melt (Table 1). On the (0 0 0 1) surface of AlB_2_, the bonding of B atoms beneath the surface Al layer is full, but those B atoms on the $\left(1 0 \overline{1 } 0\right)$, $\left(1 0 \overline{1 } 1\right)$ and $\left(1 0 \overline{1 } 2\right)$ surfaces are not fully bonded. These non-fully bonded B atoms have the opportunities to interreact with the segregated atoms at the interface.

Examination was particularly focused on the chemistry and structure of interfacial layer on the $\left(1 0 \overline{1 } 0\right)$ AlB_2_ surface, because $\left(1 0 \overline{1 } 1\right)$ and $\left(1 0 \overline{1 } 2\right)$ planes present only as the terminated plane in small scale during the growth transition of the AlB_2_ particles. As shown in Figs. 2, 3, 4, the segregation 2DC layers on $\left(1 0 \overline{1 } 0\right)$ AlB_2_ have the unique characteristics: (1) it is an atomic monolayer; (2) it is a 2-dimensional compound in which the atoms have a zigzag arrangement; and (3) it contains one or more elements according to the EELS mapping (Fig. 2). Based on the EDS mapping across the Al/AlB_2_ interfaces, the possible elements in the segregation layers include Fe, Al, Si, Mn and Ni. However, Mn and Ni are ruled out by the EDS results.

The structure and chemistry of the segregation 2DC are tentatively assessed based on various AlFeSi compounds in the ICSD database. It is found that, in most of AlFeSi compounds^15–19^, Si atoms share their sites with Al atoms, such as θ-Al_13_Fe_4_. Only in a few of AlFeSi compounds like δ-Al_4_FeSi_2_^20^, Si has the atomic sites with 100% occupancy. However, the atomic sites of Fe in most AlFeSi compounds are shared with neither Al nor Si atoms. Therefore, definition of the structure of the segregation 2DC has to consider both the AlFeSi and AlFe compounds.

Figure 4b shows the segregation layers/2DC et al./$\left(1 0\overline{ 1 } 0\right)$ AlB_2_ when viewed along the $\left[1 1 \overline{2 } 0\right]$ direction of AlB_2_ particle. The contrast of the 2DC is slightly brighter than that in the AlB_2_ particle. There is no repeated pattern with brighter and darker atoms similar to that of Al_3_Ti 2DC^29^ and Al_3_Zr 2DC^46^, indicating that Fe atoms in the segregation 2DC are evenly distributed when the 2DC is viewed along the $\left[1 1 \overline{2 } 0\right]$ direction of the AlB_2_ particle. The spacing between the first layer of the 2DC to the top Al layer of AlB_2_ is measured as 1.7 ± 0.05 Å. The spacing between the first layer and the second layer of the 2DC is 1.5 ± 0.05 Å, making the spacing from the terminated $\left(1 0\overline{ 1 } 0\right)$ plane of AlB_2_ to the second layer of 2DC be 3.2 Å, larger than 2.6 Å of the d spacing of $\left(1 0\overline{ 1 } 0\right)$ AlB_2_ planes. On the other hand, the segregation layers coherently match the $\left(1 0\overline{ 1 } 0\right)$ AlB_2_ plane, and therefore the spacing between the columns of the interfacial layer is 3.2 Å along [0 0 0 1] AlB_2_ direction, exactly the same as the d-spacing of (0 0 0 1) AlB_2_.

**Figure 10:**
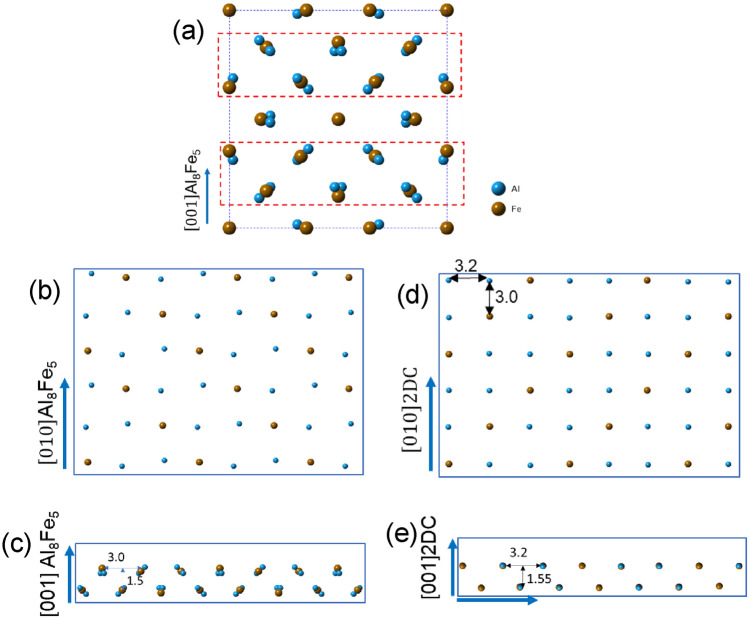
(**a**) Unit cell of bulk Al_8_Fe_5_ showing higher Fe concentration in the top, bottom and middle (001) layers, and lower and evenly distributed Fe in the building blocks (marked with red dotted frames); (**b**) projection of (001) plane (one layer) in 2DC building block of Al_8_Fe_5_, and (**c**) side view of the 2DC block (two layers) of Al_8_Fe_5_; (**d**) projection of (001) plane (one layer) of 2DC pseudo Al_8_Fe_5_, and (**e**) side view of the 2DC pseudo Al_8_Fe_5_(two layers).

The structure and chemistry of the 2DC AlFe(Si) at the Al/$\left(1 0 \overline{1 } 0\right)$ AlB_2_ interface have the 3 features: (1) a zigzag atomic arrangement along a certain direction; (2) every atomic column of the layers contains even Al/(Al + Si) + Fe atoms when viewed along the $\left[1 1 \overline{2 } 0\right]$ direction of the AlB_2_ particle; and (3) the planar spacing in the 2DC is 3.2 Å. Among various AlFe and AlFeSi compounds in the ICSD database, a high-temperature Ɛ-Al_8_Fe_5_ phase (ICSD CollCode165163) was found to meet all of the features, although a slight adjustment in the lattice parameters is needed. The Ɛ-Al_8_Fe_5_ phase is of a body-centred cubic (bcc) structure (Hume-Rothery Cu_5_Zn_8_-type, (space group *I* $\overline{4 }$ 3* m* (No. 217), *Z* = 4, Pearson symbol *cI*52, Strukturbericht designation *D*8_2_)) with the lattice parameter being a = 8.9757(2) Å^47^. In its unit cell shown in Fig. 10a, there are atomic layers with different atomic arrangement and Fe concentration. The top, middle and bottom parts have the equal atomic arrangement with higher Fe concentration than that of the building blocks (marked with red dotted frames). These layers are thereafter called higher Fe concentration layer (HFL) in this paper. It also shows that there are two repeated building blocks in the unit cell, as marked by the red dotted rectangles in Fig. 10a. In each of the building blocks, there are two layers where the atomic arrangement is close to a zigzag fashion when viewed in [1 0 0] direction of Ɛ-Al_8_Fe_5_ phase. The atomic ratio of Al and Fe for every atomic column in this direction is 2:1, i.e., Al and Fe atoms distribute evenly in the columns.

Figures 10 b-c show the projection (Fig. 10b) and the side view (Fig. 10c) of the (0 0 1) plane of the 2DC building block. It can be seen from Fig. 10b that, when viewed along the [0 1 0] direction, the atomic ratio of Al to Fe is the same as 2:1. The planar spacings ((0 0 1) and (1 0 0) planes) of the 2DC building block are 3.0 Å and 1.5 Å respectively, close to experimentally measured values 3.2 and 1.5 Å of the 2DC segregation on AlB_2_ surface. It is noted that the atomic positions of Al and Fe in the unit cell are slightly off a straight line.

Fe interfacial segregation occurs at high temperature to form the specific 2DC consisting of a couple of atomic layers. It is reasonable to believe that the positions of Al and Fe atoms at the Al/AlB_2_ interface would be relaxed due to the structural templating effect of the AlB_2_ surface. In this way, the Fe-rich segregation layer will be most likely of the crystal structure schematically shown in Fig. 10d, where Al and Fe atoms would align their positions slightly to match coherently with the Al atoms of the $\left(1 0 \overline{1 } 0\right)$ AlB_2_ plane, as shown in Fig. 10d,e. This Al_8_Fe_5_ 2DC shown in Fig. 10d,e assembles the structure and chemistry of the Fe-rich segregation layers experimentally observed on the prismatic surface of AlB_2_ (Fig. 4b).

**Figure 11:**
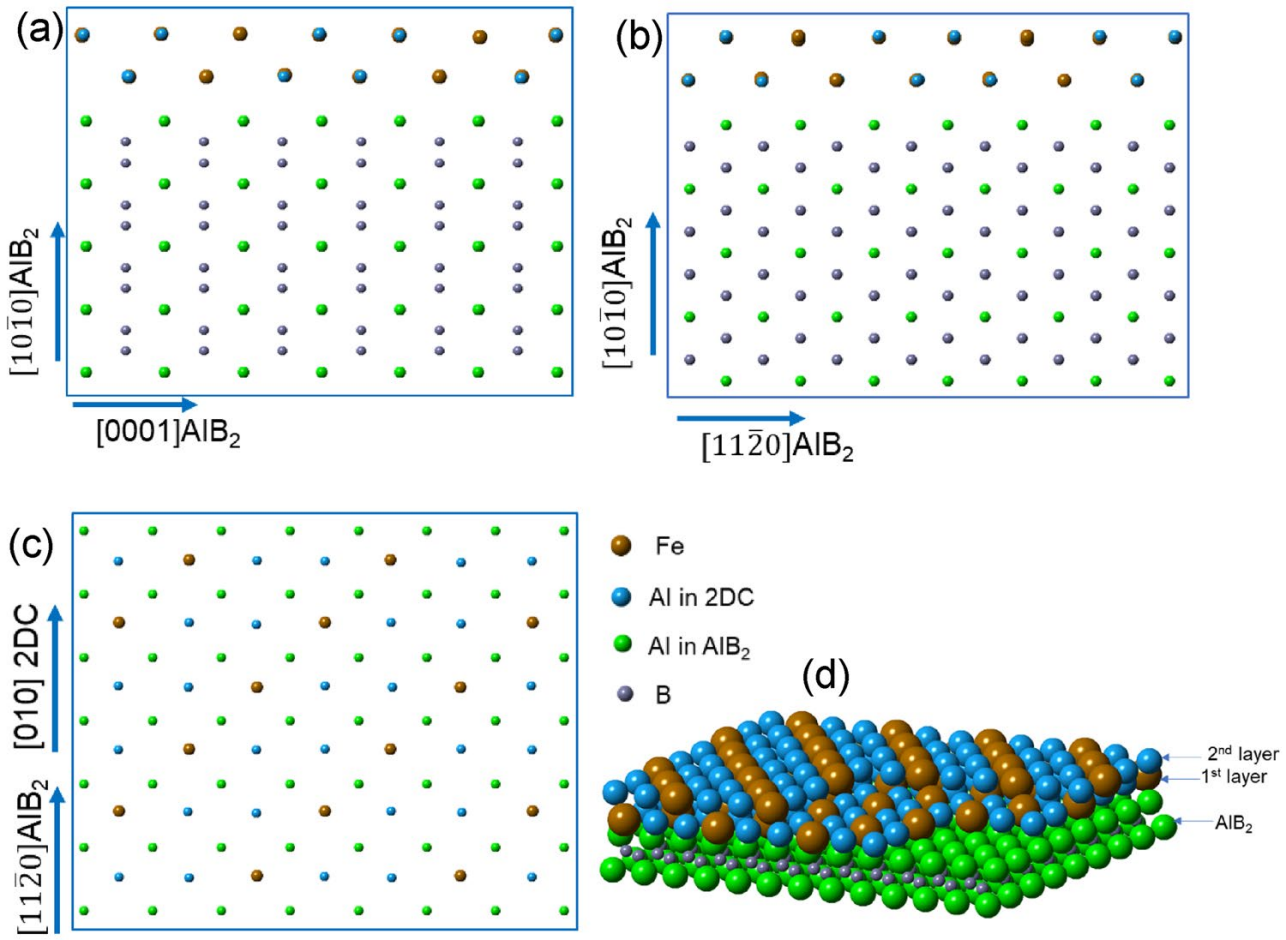
Schematic illustration showing (**a**) the Al_8_Fe_5_ 2DC on the top of the $\left(1 0 \overline{1 } 0\right)$ AlB_2_ when viewed along the $\left[1 1 \overline{2 } 0\right]$ zone direction of AlB_2_, (**b**) the Al_8_Fe_5_ 2DC on the top of the $\left(1 0 \overline{1 } 0\right)$ AlB_2_ when viewed along the $\left[0 0 0 1\right]$ zone direction of AlB_2_, (**c**) the planar matching between the first layer of 2DC and the top Al layer at $\left(1 0 \overline{1 } 0\right)$ AlB_2_, and (d) the 3D construction of the Al_8_Fe_5_ 2DC on the top of the AlB_2_ according to the OR: $\left(1 0 \overline{1 } 0\right)\left[1 1 \overline{2 } 0\right]$ AlB_2_// (0 0 1) [1 0 0] Al_8_Fe_5_ 2DC.

Figure 11a schematically shows the lattice matching between the Al_8_Fe_5_ 2DC and AlB_2_ when viewed along the $\left[1 1 \overline{2 } 0\right]$ direction of AlB_2_ and the [1 0 0] direction of the Al_8_Fe_5_ 2DC according to the OR below:

OR3 $$\left( {1\,0\,\overline{1}\,0} \right)\,\left[ {1\,1\,\overline{2}\,0} \right]\,{\text{AlB}}_{{2}} //{\text{2DC }}\left( {0 \, 0{ 1}} \right) \, \left[ {{1 }0 \, 0} \right]{\text{Al}}_{{8}} {\text{Fe}}_{{5}} {\text{2DC}},$$

In agreement with the experimental observation in Fig. 4a. It is a completely coherent match between the AlB_2_ and the pseudo Al_8_Fe_5_ 2DC. When this matching is observed from [0 0 0 1] AlB_2_ direction, i.e., by tilting 90º from Fig. 11a to b, a well-defined OR4 is revealed as

OR4 $$\left( {1\,0\,\overline{1}\,0} \right)\,\left[ {0\,0\,0\,1} \right]\,{\text{AlB}}_{{2}} //{\text{2DC }}\left( {1 \, 0{ 0}} \right) \, \left[ {{0 }0 \, 1} \right]{\text{Al}}_{{8}} {\text{Fe}}_{{5}} {\text{2DC}}.$$

Every atomic column along the direction of $\left[0 0 0 1\right]$ AlB_2_ or [001] Al_8_Fe_5_ 2DC has the same Al:Fe ratio (2:1), indicating that a uniform brightness contrast for the atomic columns of the segregation layers will appear in a HAADF image. Figure 11c shows the planar matching between the top Al layer of $\left(1 0 \overline{1 } 0\right)$ AlB_2_ planes and the first layer of Al_8_Fe_5_ 2DC.

Figure 11d schematically illustrates the 3D construction of the Al_8_Fe_5_ 2DC on the top of the $\left(1 0 \overline{1 } 0\right)$ AlB_2_ surface. It is seen that although there are some pure Fe columns, when viewed along the lower zone axis of AlB_2_ such as $\left[0 0 0 1\right]$ or $\left[1 1 \overline{2 } 0\right]$, the pure Fe column can’t be viewed. This can explain the result in Fig. 4 that the newly formed 2DC is evenly brighter than AlB_2_ but no bright and dark pattern.

**Figure 12:**
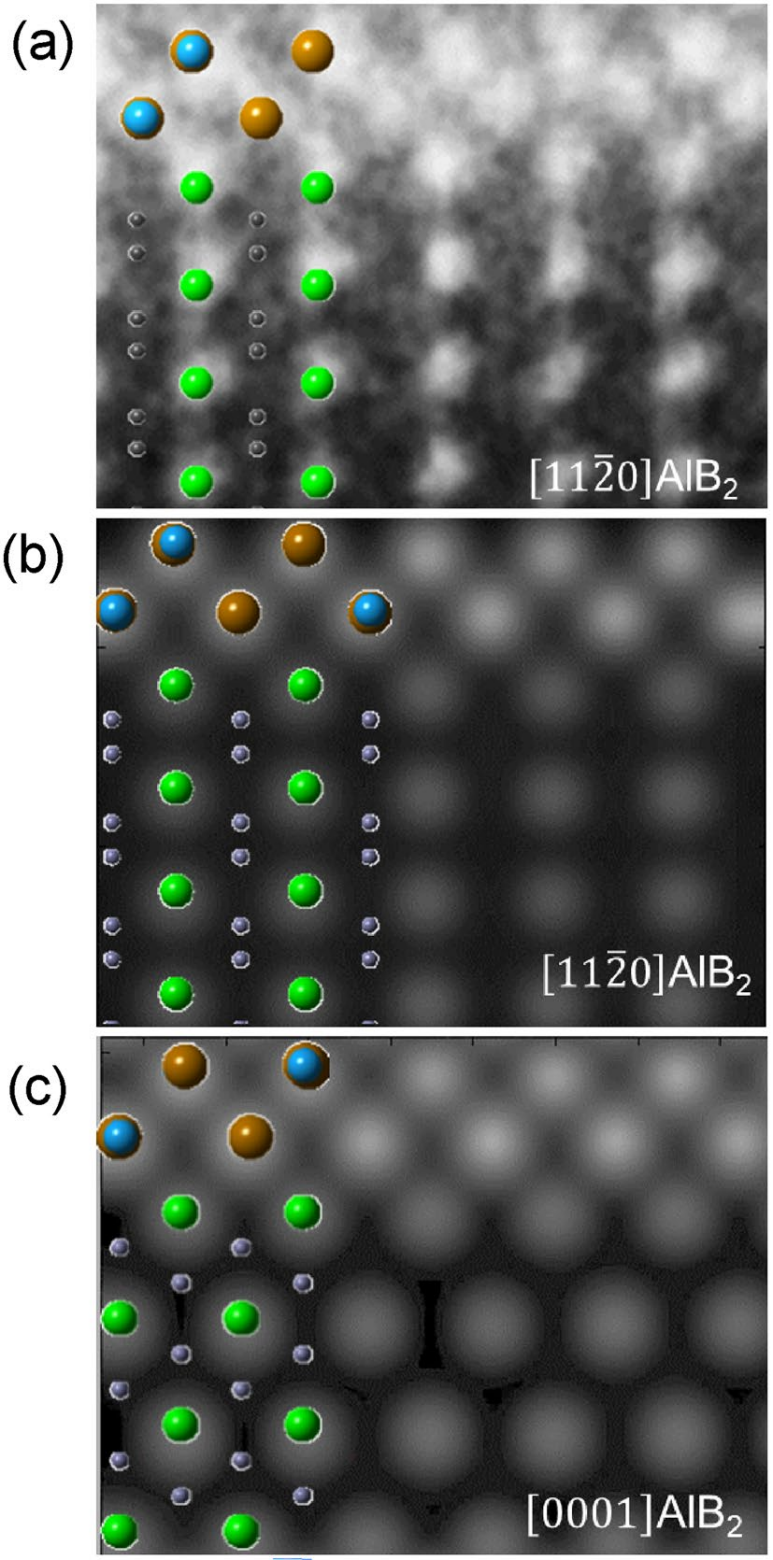
(**a**) Experimental STEM HADDF image with the schematically illustration viewed along the $\left[1 1 \overline{2 } 0\right]$ of the AlB_2_, (**b**,**c**) the simulated STEM HAADF images viewed along (**b**) $\left[1 1 \overline{2 } 0\right]$, and (**c**) $\left[0 0 0 1\right]$ of AlB_2_. The simulation of STEM HAADF was carried out using the QSTEM multi-slice simulation package developed by Koch^41^, according to the constructed Al_8_Fe_5_ 2DC on the surface of the $\left(1 0 \overline{1 } 0\right)$ of the AlB_2_.

Simulation of STEM HAADF images was performed using the QSTEM multi-slice simulation package developed by Koch^41^, in order to validate the established characteristics of the segregation layers. It is shown that the experimental HADDF image viewed along the $\left[1 1 \overline{2 } 0\right]$ AlB_2_ direction in Fig. 12a is consistent with the simulated HADDF image in Fig. 12b. Figure 12c shows the simulated STEM HAADF image along the $\left[0 0 0 1\right]$ of the AlB_2_ using the model of Fig. 11b. It is clear that the segregation 2DC et al./$\left(1 0 \overline{1 } 0\right)$ AlB_2_ is in-planar ordered when viewed along the $\left[0 0 0 1\right]$ direction and has a similar zigzag pattern in the atomic arrangement and higher brightness than that in the AlB_2_ also.

### Composition templating for heterogeneous nucleation of α-Al_15_(Fe, Mn)_3_Si_2_

Previous work^29,46,48^ has demonstrated that interfacial segregation of alloy elements on nucleation substrates and the resultant formation of 2DC or 2DS such as Al_3_Ti 2DC and Al_3_Zr 2DC on top of (0 0 0 1) TiB_2_ particles change the nucleation potency of the substrates and thus affect the heterogeneous nucleation process correspondingly. For example, Al_3_Ti 2DC on TiB_2_ and enhances heterogeneous nucleation of α-Al. However, the formation Al_3_Zr 2DC and Si 2DS destroyed the Al_3_Ti 2DC, therefore poisoned the TiB_2_ and impeded nucleation of α-Al.

In this study, AlB_2_ particles in Al-2.8Ti-1.8B-1.0Fe master alloy were modified by Fe interfacial segregation on the $\left(1 0 \overline{1 } 0\right)$ planes, leading to the changes in not only the structure from AlB_2_ to a new Al_8_Fe_5_ 2DC (structural templating), but also the chemistry from Fe-free to Fe-rich (compositional templating) of the AlB_2_ particles as the nucleation substrates. It is revealed that heterogenous nucleation of α-Al_15_(Fe, Mn)_3_Si_2_ takes place on the $\left(1 0 \overline{1 } 0\right)$ AlB_2_ surface with a well-defined OR (OR2). In fact, heterogeneous nucleation of the α-Al_15_(Fe, Mn)_3_Si_2_ occurs actually on the Al_8_Fe_5_ 2DC which covered at $\left(1 0 \overline{1 } 0\right)$ AlB_2_ surface.

As discussed above, the Al_8_Fe_5_ 2DC segregation layer has the OR with AlB_2_ as OR 3:$\left(1 0 \overline{1 } 0\right)\left[1 1 \overline{2 } 0\right]$ AlB_2_// (0 0 1) [1 0 0] Al_8_Fe_5_ 2DC. Therefore, the OR (OR5) between the Al_8_Fe_5_ 2DC and the α-Al_15_(Fe, Mn)_3_Si_2_ can be worked out from OR2 as below:

OR5 $$\left( {0 \, 0{ 1}} \right) \, \left[ {{1 }0 \, 0} \right]{\text{ Al}}_{{8}} {\text{Fe}}_{{5}} {\text{2DC }}// \, \left( {0 \, 0{ 1}} \right) \, \left[ {0{ 1 1}} \right]\alpha - {\text{Al}}_{{{15}}} \left( {{\text{Fe}},{\text{ Mn}}} \right)_{{3}} {\text{Si}}_{{2}} .$$

**Figure 13:**
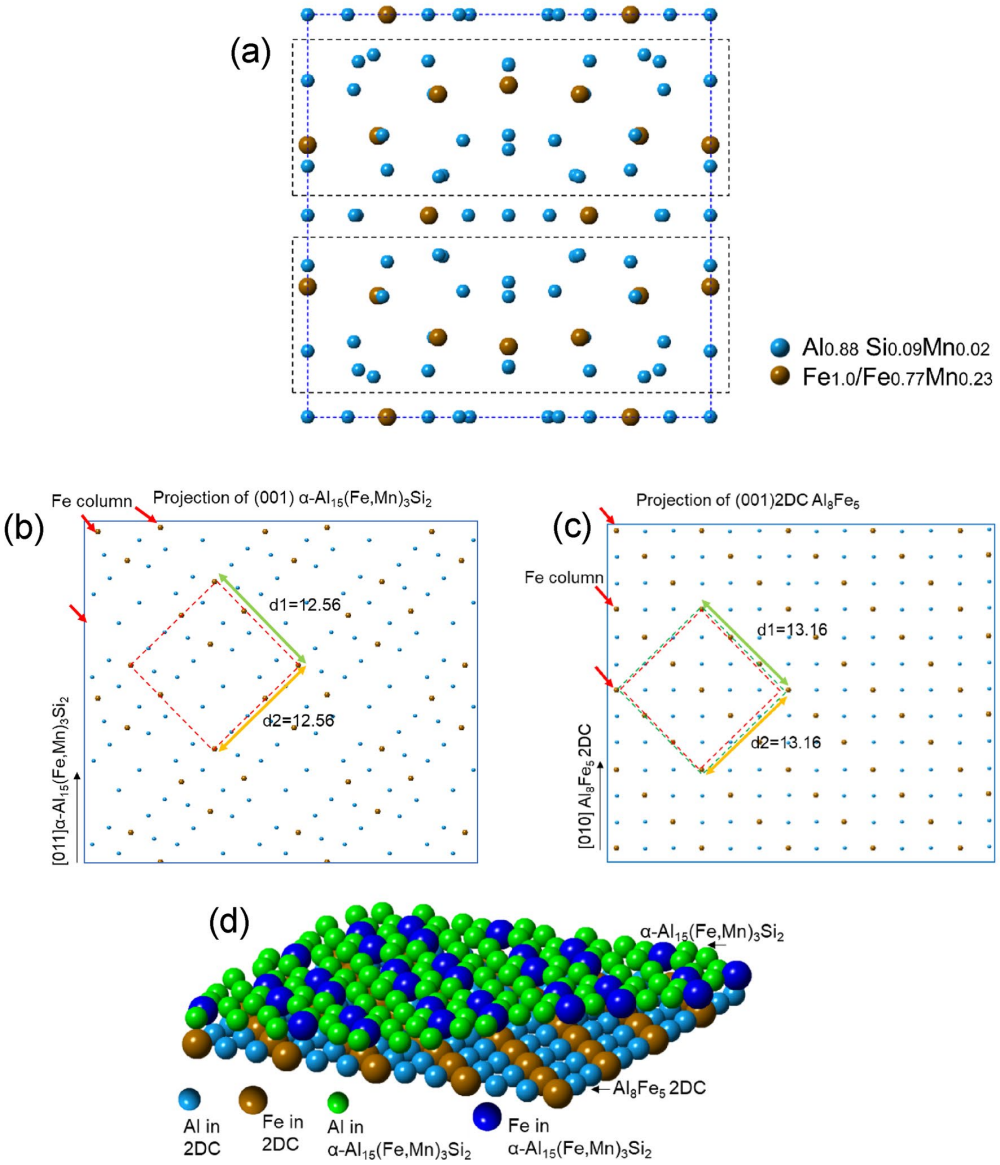
(**a**) Unit cell of α-Al_15_(Fe, Mn)_3_Si_2_ previewed from [1 0 0] direction showing flat layers in the top, middle and bottom (0 0 1) layers, and two equal building blocks (marked with black dotted frames) inside of the bulk, (**b**) the projection of (0 0 1) plane of α-Al_15_(Fe, Mn)_3_Si_2_ with the flat layer showing regular Fe columns with minimum repeat unit (marked with red dotted square frame) with d = 12.56 Å, (**c**) one (001) layer from the building block of Al_8_Fe_5_ showing the regularly repeatable Fe columns (marked with green dotted square frame) with the d = 13.16 Å which can corresponding to the unit of α, and (**d**) the 3D construction of the α-Al_15_(Fe, Mn)_3_Si_2_ on the top of the Al_8_Fe_5_ 2DC according to the OR: (0 0 1)[1 1 0] α-Al_15_(Fe, Mn)_3_Si_2_ // (0 0 1)[1 0 0] Al_8_Fe_5_ 2DC.

α-Al_15_(Fe, Mn)_3_Si_2_ has a bcc structure with the lattice parameter of *a* = 12.56 Å^47^. It contains 138 atoms in the unit cell, as shown in Fig. 13a. The α-Al_15_(Fe, Mn)_3_Si_2_ bulk phase contains the equal flat layers at the top, middle and bottom layers and some building blocks inside of the bulk. The flat layer is considered the firstly templating layer on the nucleation substrates/segregation Al_8_Fe_5_ 2DC due to the lower formation energy. The projections of (0 0 1) α-Al_15_(Fe, Mn)_3_Si_2_ (top layer) and (0 0 1) of Al_8_Fe_5_ 2DC are displayed in Fig. 13b and c, respectively. Figure 13b shows that the flat layer of the (0 0 1) planes of α-Al_15_(Fe, Mn)_3_Si_2_ is more closely packed than that in the building blocks. Fe columns in Fig. 13b are repeated with a minimum unit (marked by red dotted frame) of 12.56 × 12.56 Å square. The projection of (0 0 1) of Al_8_Fe_5_ 2DC in Fig. 13c reveals that regular Fe column distribution as α-Al_15_(Fe, Mn)_3_Si_2_. The minimum matching Fe unit square frame (marked in green) is 13.16 × 13.16 Å square.

The lattice misfit (*f*_1_) between the α-Al_15_(Fe, Mn)_3_Si_2_ and the Al_8_Fe_5_ 2DC along the Fe columns is calculated as -4.7% according to the epitaxial nucleation theory^39^. The misfit (*f*_2_) between the α-Al_15_(Fe, Mn)_3_Si_2_ and the AlB_2_ along the Fe columns according to OR2 is calculated as -5.7%. The difference between *f*_1_ and *f*_2_ is small, indicating that the structural templating provided by the modified AlB_2_ particles caused by the interfacial segregation of Fe on the $\left(1 0 \overline{1 } 0\right)$ surface contributes less than the compositional templating to the enhanced heterogeneous nucleation of α-Al_15_(Fe, Mn)_3_Si_2_. In other words, heterogeneous nucleation of α-Al_15_(Fe, Mn)_3_Si_2_ is enhanced mainly by the composition templating (Fe columns) provided by the interfacial segregation of Fe-rich layers on the $\left(1 0 \overline{1 } 0\right)$ AlB_2_. Figure 13d shows the 3D construction of the α-Al_15_(Fe, Mn)_3_Si_2_ on the top of the Al_8_Fe_5_ 2DC according to the OR5. It is clear that the Al_8_Fe_5_ 2DC provides the exact composition templating required for heterogeneous nucleation of α-Al_15_(Fe, Mn)_3_Si_2_.

Interfacial segregation of Si at the Al/ $\left(1 0 \overline{1 } 0\right)$ AlB_2_ interface also contributes to the enhancement of heterogeneous nucleation of FIMCs by providing Si atoms. Si atomic positions in either Al_8_Fe_5_ 2DC or α-Al_15_(Fe, Mn)_3_Si_2_ are shared with Al with varying occupancy. The concentration of Si on the templating layers is also variable. In contrast, heterogeneous nucleation of Fe-rich IMCs requiring Fe atoms in specific atomic positions is much difficult than that of Si and Al atoms.

Four elements Al, Fe, Mn and Si are required for the formation of α-Al_15_(Fe, Mn)_3_Si_2_. The composition and structural templating of Al, Fe and Si can be provided by the modified AlB_2_ as discussed earlier. In crystal structure of α-AlFeMnSi^18^, Mn shares the atomic sites with all Al sites with 0.02 occupancy and half Fe sites with 0.23 occupancy. The atomic position for Mn is much flexible than that of Fe and the Mn concentration can be variable in a large range^14^, indicating the less important of the effects of Mn templating on the heterogeneous nucleation of α-Al_15_(Fe, Mn)_3_Si_2_. This means that composition templating and structural templating of Al and Fe are critical, whilst the templating of Mn and Si being less important comparatively.

Current understanding of heterogeneous nucleation is mainly based on the structural templating for pure metal or solid solution, which emphasizes the lattice misfit at the interface between the nucleating solid and substrates. Heterogeneous nucleation of intermetallic compounds is inherently more difficult, with the requirements of both the creation of a crystal structure and the positioning of 2 or more types of elements in the lattice with specified compositions. This makes composition templating a very important factor for heterogeneous nucleation of IMCs in addition to structural templating.

In this work we have demonstrated theoretically and experimentally that providing Fe and Si composition templating is an effective approach to significantly enhance heterogeneous nucleation of Fe-IMCs, although heterogeneous nucleation of the IMCs requires an undercooling few tens of degree Kelvin, which is more than an order of magnitude higher than that for pure metals. A technique has been developed in this study to achieve the deliberate segregation of the key constitution element Fe on the potential nucleation substrates AlB_2_ particles in a master alloy. Fe and Si co-segregation on the $\left(1 0 \overline{1 } 0\right)$ AlB_2_ surfaces leads to the formation of a zigzag Ɛ-Al_8_Fe_5_ 2DC, which provided both the structural templating and the compositional templating for heterogeneous nucleation of α-Al_15_(Fe, Mn)_3_Si_2_. The Ɛ-Al_8_Fe_5_ 2DC provides Fe columns with the right positioning required by creating the structure of α-Al_15_(Fe, Mn)_3_Si_2_. Compared to the small decrease misfit from − 5.7 to − 4.7% due to the newly formed 2DC segregation layers, the contribution of the composition templating is more significant for the enhanced heterogeneous nucleation of the α-Al_15_(Fe, Mn)_3_Si_2_. The modified AlB_2_ (Fe) serves as the potent nucleation substrate in terms of structure and chemistry, leading to the greatly refined primary α-Al_15_(Fe, Mn)_3_Si_2_ particles with considerably increased number density.

Overall, composition templating as important part as structural templating for the heterogeneous nucleation of intermetallic compounds has been proposed and validated. Providing composition templating as an effective approach to achieving significant refinement of Fe-rich IMCs in Al alloys has been developed in this work, which is applicable for not only Fe-rich intermetallic compound in Al alloys, but also for all compounds in metallic materials.

## Summary

1. Hybrid (Al, Ti)B_2_ particles were synthesized in an Al-2.8Ti-1.8B master alloy (with 0.52wt.% excess B), with AlB_2_ covering the surface of the hybrid particles. Fe was allowed to segregate at the Al/$\left(1 0 \overline{1 } 0\right)$ AlB_2_, Al/$\left(1 0 \overline{1 } 1\right)$ AlB_2_ and Al/$\left(1 0 \overline{1 } 2\right)$ AlB_2_ interfaces in an Al-2.8Ti-1.8B master alloy with 1 wt.% Fe addition and holding at 800 ℃ for 4 h. No Fe segregation was observed on $\left(0 0 0 1\right)$ plane of AlB_2_.
2. Si at impurity level (0.19 wt.%) was found to co-segregate with Fe on all of the surface of the hybrid (Al, Ti)B_2_ particles in the Al-2.8Ti-1.78B-1Fe master alloy.
3. Interfacial segregation of Fe et al./$\left(1 0 \overline{1 } 0\right)$ AlB_2_ interface leads to the formation of the zigzag 2DC arrangement and the segregation 2DC layer was further identified as a 2D layer of the Pseudo Ɛ-Al_8_Fe_5_ phase which has a body-centred cubic structure of the Hume-Rothery Cu_5_Zn_8_-type with a lattice parameter of 8.9757(2) Å at 1120 ℃, consistent with the STEM HAADF simulation results.
4. The AlB_2_ particles with Fe interfacial segregation heterogeneously nucleates α-Al_15_(Fe, Mn)_3_Si_2_ phase, resulting in a well-defined orientation relationship between the α-Al_15_(Fe, Mn)_3_Si_2_ and the embedded AlB_2_ particle: $\left(0 0 0 1\right)$ AlB_2_ // $\left(0 1 \overline{1 }\right)$ α-Al_15_(Fe, Mn)_3_Si_2_ and $\left[1 0 \overline{1 } 0\right]$ AlB_2_ // 4.5° [1 1 1] α-Al_15_(Fe, Mn)_3_Si_2_, equivalent to the OR: $\left(1 0 \overline{1 } 0\right)$ AlB_2_ // (0 0 1) α-Al_15_(Fe, Mn)_3_Si_2_, and $\left[1 1 \overline{2 } 0\right]$ AlB_2_ // [0 1 1] α-Al_15_(Fe, Mn)_3_Si_2_.
5. Quantification assessment by the casting experiment confirmed that significant refinement of the primary α-Al_15_(Fe, Mn)_3_Si_2_ intermetallic was achieved by the introduction of the Al-2.8Ti-1.78B-1Fe master alloy containing AlB_2_ particles which have Fe segregation at the Al/$\left(1 0 \overline{1 } 0\right)$ AlB_2_ interface, containing resulted in the grain with the average size decreasing from 38.7 ± 6.8 μm to 19.2 ± 5.6 μm.

## Data Availability

The datasets used and/or analysed during the current study available from the corresponding author on reasonable request. All data generated or analysed during this study are included in this published article.
